# Longitudinal effects of prenatal exposure to plastic‐derived chemicals and their metabolites on asthma and lung function from childhood into adulthood

**DOI:** 10.1111/resp.14386

**Published:** 2022-10-02

**Authors:** Rachel E. Foong, Peter Franklin, Francesca Sanna, Graham L. Hall, Peter D. Sly, Eric B. Thorstensen, Dorota A. Doherty, Jeffrey A. Keelan, Roger J. Hart

**Affiliations:** ^1^ Wal‐Yan Centre for Respiratory Research Telethon Kids Institute Perth Western Australia Australia; ^2^ School of Allied Health Curtin University Perth Western Australia Australia; ^3^ School of Population Health University of Western Australia Perth Western Australia Australia; ^4^ Child Health Research Centre The University of Queensland South Brisbane Queensland Australia; ^5^ Liggins Institute, University of Auckland Auckland New Zealand; ^6^ Women and Infants Research Foundation King Edward Memorial Hospital Perth Western Australia Australia; ^7^ Division of Obstetrics and Gynaecology University of Western Australia Perth Western Australia Australia; ^8^ School of Biomedical Sciences University of Western Australia Perth Western Australia Australia

**Keywords:** asthma, environmental exposure, longitudinal birth cohort study, lung function, pregnancy, the Raine study

## Abstract

**Background and Objective:**

Environmental exposure to phthalates and bisphenol A (BPA), chemicals used in the production of plastics, may increase risk for asthma and allergies. However, little is known about the long‐term effects of early life exposure to these compounds. We investigated if prenatal exposure to these compounds was associated with asthma, allergy and lung function outcomes from early childhood into adulthood in a cohort study.

**Methods:**

Maternal serum samples collected from 846 pregnant women in the Raine Study were assayed for BPA and phthalate metabolites. The children of these women were followed up at 5, 13 and 22 years where spirometry and respiratory questionnaires were conducted to determine asthma and allergy status. Lung function trajectories were derived from longitudinal spirometry measurements. Multinomial logistic regression and weighted quantile sum regression was used to test associations of individual and chemical mixtures with asthma phenotypes and lung function trajectories.

**Results:**

Effects of prenatal BPA and phthalates on asthma phenotypes were seen in male offspring, where BPA was associated with increased risk for persistent asthma, while mono‐iso‐butyl phthalate and mono‐iso‐decyl phthalate was associated with increased risk for adult asthma. Prenatal BPA had no effect on lung function trajectories, but prenatal phthalate exposure was associated with improved lung function.

**Conclusion:**

Prenatal BPA exposure was associated with increased likelihood of persistent asthma in males, while prenatal phthalate exposure was associated with increased likelihood of adult asthma in males. Results suggest that prenatal exposure to prenatal BPA and phthalates affect asthma risk, particularly in males, however lung function was not adversely affected.

## Graphical abstract

This is the first study to show that prenatal exposure to plastic‐derived chemicals and their metabolites may increase asthma risk and alter lung function from childhood into adulthood. These chemicals increased risk of asthma in males, but did not adversely affect lung function.

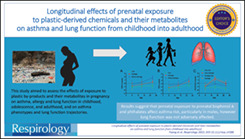

**See related** Editorial

## INTRODUCTION

The production of plastic is associated with the release of chemical additives such as bisphenol A (BPA) and phthalates. Bisphenols are used in the manufacture of polyvinyl chloride (PVC) and often found in children's toys, food and drink containers and the inner coating of cans and bottles.^1^ Similarly, phthalates are plasticizers also used in the manufacture of PVC found in building materials and can also be found in food packaging, low molecular weight phthalates are also commonly found in personal care products and cosmetics.^2^ Given their widespread use, these chemicals are ubiquitous in the developed world.

Biomarkers of BPA and phthalate metabolites have been measured in children and pregnant women.^2^, ^3^ BPA and phthalates can cross the placental barrier and exposure prenatally may have a greater impact on the immune system compared to exposure later in life.^4^ The effects of BPA and phthalate exposure on the immune system, potentially via endocrine disruption and adverse effects on fetal lung development, have been hypothesized to be the mechanistic links to increased risk of asthma and allergies in children.^2^

Birth cohort studies have examined associations between prenatal BPA and phthalate exposure with asthma and allergic diseases. The findings of most, but not all, support a link with an increased risk for asthma and allergies.^2^, ^4^, ^5^ Moreover, the effects of these chemicals on long‐term respiratory health have yet to be fully determined. To date, the effects of prenatal exposure have been mostly studied in children aged up to 12 years,^6^ with no studies extending beyond adolescence and into adulthood.

Given that early‐life environmental exposures may alter longitudinal lung function and respiratory phenotypes,^7^ this study hypothesizes that prenatal BPA and phthalate exposure is associated with increased risk for asthma and respiratory health impairment in children from early childhood into adulthood. We aimed to assess the effects of exposure to plastic by‐products and their metabolites in pregnancy on asthma, allergy and lung function in childhood, adolescence and adulthood, and on asthma phenotypes and lung function trajectories.

## METHODS

### Study population

The Raine Study is a longitudinal birth cohort where between 1989 and 1991, 2900 pregnant women were recruited into the study at King Edward Memorial Maternity Hospital in Perth, Western Australia.^8^, ^9^ Detailed respiratory assessment were conducted at the 5, 13 and 22‐year follow‐ups.^10^, ^11^, ^12^ Participants with matched maternal samples with BPA or phthalates measurements at 18‐ and 34‐weeks' gestation included in this study (Figure S1 in the Supporting Information).

### BPA and phthalate metabolite measurement

Maternal serum samples in 200 μl aliquots were pooled for analysis of BPA and phthalate metabolites to provide an estimate of exposure throughout gestation. Prior to analyses, pilot studies were conducted to ensure that sample collection and storage were valid for measurement of phthalates and BPA.^13^, ^14^

Total BPA was measured by liquid chromatography–mass spectrometry (LC/MS) after deconjugation.^14^ Blanks were included throughout the deconjugation, extraction and analysis steps to exclude external contamination; BPA levels were below the limit of detection (LOD) of 0.005 μg/L in blanks.

Eleven phthalate metabolites: mono‐ethyl phthalate (MEP), mono‐*n*‐butyl phthalate (MnBP), mono‐iso‐butyl phthalate (MiBP), mono‐benzyl phthalate (MBzP), mono‐*n*‐pentyl phthalate (MPP), mono‐(2‐ethyl‐hexyl) phthalate (MEHP), mono(2‐ethyl‐5‐carboxypentyl) phthalate (MECPP), mono‐octyl phthalate (MOP), mono‐iso‐nonyl phthalate (MiNP), mono‐carboxyiso‐nonyl phthalate (MCiOP) and mono‐iso‐decyl phthalate (MiDP) were measured by isotope‐diluted liquid chromatography–tandem mass spectrometry (LC–MS/MS), after deconjugation.^13^ MEHP and MECPP were combined and expressed as the sum of di‐[2‐ethylhexyl] phthalate (DEHP) metabolites (∑DEHP), and MiNP and MCiOP combined and expressed as the sum of di‐iso‐nonyl phthalate (DiNP) metabolites (∑DiNP). Further methods are detailed in Appendix S1 in the Supporting Information.

#### Respiratory outcomes and co‐variates

Current asthma was defined as a positive response to two of the following criteria, (i) Doctor diagnosis of asthma ever; (ii) Medications for asthma treatment during the last 12 months and (iii) Wheezing in the past 12 months. Asthma phenotypes were defined as (1) early‐onset, (2) late onset or (3) persistent, where it was termed early‐onset if asthma was present at 6 years of age only; late‐onset if asthma occurred at 14 or 22 years of age, but not earlier; and persistent if asthma was present from childhood up to young adulthood.

Allergic sensitization was assessed in participants by skin prick testing (SPT) with details on specific allergens in Appendix S1 in the Supporting Information.

Spirometry was conducted according to the American Thoracic Society in place at each follow‐up.^15^ Reference values were derived from the Global Lung Function Initiative (GLI) reference equations^16^ with forced expiratory volume in 1 s (FEV_1_), forced vital capacity (FVC) and ratio of the forced expiratory volume in 1 s to the forced vital capacity (FEV_1_/FVC) reported as outcome measures. All analyses with lung function outcomes were conducted using GLI z‐scores.

Potential confounders included: (i) quartiles of predicted equivalized total disposable household income or reported household income; (ii) maternal smoking during pregnancy; (iii) maternal age at birth and (iv) ever breastfed. Further detail on identification of these confounders is in Appendix S1 in the Supporting Information.

### Statistical analysis

Random effects logistic regression models were used to examine the associations between BPA and phthalates with repeated binary asthma and allergy outcomes. Linear mixed‐effects models with random intercepts for individual participants were used to determine effects of prenatal exposures with repeated spirometry outcomes. Lung function trajectories were plotted^17^ using group‐based trajectory modelling (Figure S2 in the Supporting Information). Multinomial logistic regression was used for associations between prenatal exposures and asthma phenotypes and lung function trajectory groups with adjustment for potential confounding (Figure S3 in the Supporting Information). Analyses were also stratified by sex. Weighted quantile sum (WQS) regression was also used to model the association of a mixture of phthalates with these outcomes. Further detail is provided in Appendix S1 in the Supporting Information. Hypothesis tests were two sided with *p*‐values of <0.05 considered statistically significant. Analyses were conducted using Stata SE 16.1 (College Station, TX) and WQS regression using the ‘gWQS’ package in R (Version 3.1.4).^18^, ^19^

## RESULTS

### Study population characteristics

Of the participants included in this study, 846 had matched maternal BPA or phthalate measurement with at least one respiratory outcome measure. Participant demographics are included in Table 1.

**TABLE 1:** Participant demographics

Characteristics							
Sex (Male)	473/846 (55.9%)						
Birth weight (kg)/(z‐scores)	Male			3.35 (0.58)/0.03 (1.31)			
	Female			3.15 (0.63)/−0.13 (1.33)			
		*N*	5 years	*N*	13 years	*N*	22 years
Height (cm)	Male	473	116.42 (4.91)	364	167.30 (8.61)	209	178.79 (8.48)
	Female	373	115.11 (5.00)	306	162.29 (6.46)	161	166.62 (6.40)
Weight (kg)	Male	473	21.61 (3.45)	364	59.22 (13.58)	209	80.63 (16.08)
	Female	373	20.89 (3.44)	306	56.68 (13.16)	161	73.87 (22.12)
Current asthma	Male	473	101/473 (21.4%)	364	75/364 (20.6%)	209	34/209 (16.3%)
	Female	373	69/373 (18.5%)	306	55/306 (18.0%)	161	34/161 (21.1%)
Allergy (positive skin prick test)	Male	354	139/354 (39.3%)	328	187/328 (57.0%)	178	131/178 (73.6%)
	Female	227	72/227 (31.7%)	203	75/203 (36.9%)	110	73/110 (66.4%)
FEV1 z‐score	Male	362	−0.85 (0.97)	313	−0.51 (1.03)	209	−0.13 (0.96)
	Female	302	−0.92 (0.97)	274	−0.54 (1.01)	124	−0.21 (0.75)
FVC z‐score	Male	361	−1.16 (1.00)	313	−0.92 (1.04)	209	−0.02 (0.92)
	Female	299	−1.18 (0.97)	274	−0.88 (0.98)	124	0.01 (0.84)
FEV/FVC z‐score	Male	251	−0.87 (0.98)	313	0.72 (1.19)	209	−0.23 (0.87)
	Female	187	−0.91 (0.94)	192	0.63 (1.04)	124	−0.34 (0.94)

*Note*: Data are presented as mean (SD) or number (percentage).

Detectable levels of BPA and phthalates were found in maternal serum in all but one participant (Table 2). Only one participant had MEHP below the LOD. The least detectable category of phthalate metabolites was MHBP with up to 66.3% of participants not having detectable levels. There was only strong correlation between MIBP and MNBP (Figure S4 in the Supporting Information).

**TABLE 2:** Concentrations of bisphenol A (BPA) and phthalates in maternal serum samples

	Limit of detection	*N* (%) < LOD	Min	25th percentile	50th percentile	75th percentile	90th percentile	Max
BPA (μg/L) *n* = 837	0.005	99 (11.8%)	0.001	0.107	0.316	0.763	1.596	12.58
∑MBP (ng/ml)			0	1.86	3.67	7.54	16.22	532.76
MiBP (ng/ml) *n* = 986	0.75	363 (36.8%)	0	0	1.19	2.22	4.33	74.04
MnBP (ng/ml) *n* = 986	0.61	102 (10.3%)	0	1.34	2.53	5.08	11.58	463.47
∑LMWP (ng/ml)			0	3.87	7.55	14.42	27.71	2572.53
MEP (ng/ml) *n* = 986	0.65	199 (20.2%)	0	0.95	2.91	6.75	14.03	2106.54
MHBP (ng/ml) *n* = 986	0.22	654 (66.3%)	0	0	0	0.29	0.45	2.53
∑HMWP (ng/ml)			1.30	8.45	11.42	14.53	17.99	81.82
MBzP (ng/ml) *n* = 986	0.26	574 (58.2%)	0	0	0	0.49	1.00	73.77
MCPP (ng/ml) *n* = 986	0.19	580 (58.8%)	0	0	0	0.35	0.66	7.58
∑DEHP (ng/ml)			1.86	6.33	8.78	11.89	16.21	64.62
MEHP (ng/ml) *n* = 986	0.74	1(0.1%)	0	2.49	3.73	5.59	7.75	32.18
MECPP (ng/ml) *n* = 986	0.25	70 (7.1%)	1.83	6.33	8.78	11.89	16.21	64.62
∑DiNP (ng/ml)			0	3.06	5.72	8.63	10.88	76.96
MiNP (ng/ml) *n* = 986	0.53	40 (4.1%)	0	2.02	3.78	5.74	7.24	53.68
MCiOP (ng/ml) *n* = 986	0.13	427 (43.3%)	0	0	0.15	0.28	0.54	15.28
MiDP (ng/ml) *n* = 986	0.72	701(71.1%)	0	0	0	0.86	2.13	21.21

Abbreviations: MBzP, mono‐benzyl phthalate; MCiOP, mono‐carboxyiso‐nonyl phthalate; MCPP, mono‐3‐carboxypropyl phthalate; MECPP, mono‐(2‐ethyl‐5‐carboxypentyl) phthalate; MEHP, mono‐(2‐ethyl‐hexyl) phthalate; MEP, mono‐ethyl phthalate; MiBP, mono‐iso‐butyl phthalate; MiDP, mono‐iso‐decyl phthalate; MiNP, mono‐iso‐nonyl phthalate; MnBP, Mono‐*n*‐butyl phthalate; MHBP, mono‐(3‐hydroxybutyl) phthalate; ∑DEHP, the sum of di‐[2‐ethylhexyl] phthalate metabolites; ∑DiNP, the sum of di‐iso‐nonyl phthalate metabolites; ∑HMWP, molar sum of MBzP, MEHP, MECPP, MCPP, MiNP, MCiOP and MiDP high molecular weight phthalates; ∑LMWP, molar sum of low molecular weight phthalates MEP, MiBP, MnBP and MHBP; ∑MBP, sum of MiBP and MnBP.

### The effects of prenatal BPA and phthalate exposure on allergy, asthma and asthma phenotypes from childhood to adulthood

Ten‐fold increases in prenatal BPA concentrations were associated with 17% increased odds of asthma in males after adjustment for potential confounders. In contrast, 10‐fold increased concentrations of prenatal MiNP were associated with almost double the odds of asthma in females (Odds ratio: 1.97, 95% CI: 1.03, 3.77). Prenatal ∑DiNP concentrations were associated with 71% increased odds for asthma in females (Figure S5A in the Supporting Information). The only associations for phthalates and their metabolites with the presence of allergies was for MEP, where in males there was 52% higher odds of allergy (Figure S5B in the Supporting Information).

Significant associations with asthma phenotypes were found in males only, where a 10‐fold increase in prenatal BPA exposure was significantly associated with a 23% higher relative risk for persistent asthma compared with no asthma (Table 3). Prenatal MBzP exposure was associated with 87% lower relative risk for persistent asthma, but with over a doubling of the relative risk ratio (RRR) for transient asthma (RRR: 2.26, 95% CI: 1.14, 4.48). Ten‐fold increases in MiBP and MiDP were associated with RRRs of 2.42 (95% CI: 1.08, 5.42) and 4.39 (95% CI: 1.16, 16.56), respectively, for adult asthma compared with no asthma. Sensitivity analyses with additional confounders did not alter results (Table S1 in the Supporting Information).

**TABLE 3:** Associations between log‐transformed bisphenol A (BPA) and phthalates with asthma phenotypes

		Relative risk ratios (95% CI); *p*‐value		
	Sex	Transient asthma (*n* = 66, 40 males and 26 females)	Adult asthma (*n* = 26, 14 males and 12 females)	Persistent asthma (*n* = 79, 44 males and 35 females)
BPA (μg/L)	All	**1.18 (1.00, 1.39); *p* = 0.046**	0.97 (0.81, 1.17); *p* = 0.79	**1.20 (1.03, 1.39), *p* = 0.018**
	Male	1.22 (0.99, 1.51); *p* = 0.054	1.24 (0.88, 1.73); *p* = 0.22	**1.23 (1.02, 1.48); *p* = 0.031**
	Female	1.13 (0.87, 1.47); *p* = 0.37	0.82 (0.65, 1.03); *p* = 0.088	1.22 (0.94, 1.58); *p* = 0.14
∑MBP (ng/ml)	All	1.30 (0.97, 1.74); *p* = 0.082	1.30 (0.87, 1.95);*p* = 0.20	0.92 (0.69, 1.22); *p* = 0.55
	Male	1.27 (0.82, 1.99); *p* = 0.29	1.58 (0.78, 3.20); *p* = 0.21	0.79 (0.50, 1.26); *p* = 0.33
	Female	1.36 (0.89, 2.07); *p* = 0.16	1.27 (0.75, 2.15); *p* = 0.37	0.97 (0.89, 1.43); *p* = 0.66
MiBP (ng/ml)	All	1.17 (0.79, 1.75); *p* = 0.43	1.44 (0.86, 2.43); *p* = 0.17	0.84 (0.57, 1.23); *p* = 0.36
	Male	1.14 (0.64, 2.02); *p* = 0.65	**2.42 (1.08, 5.42); *p* = 0.032**	0.87 (0.50, 1.52); *p* = 0.63
	Female	1.22 (0.68, 2.17); *p* = 0.51	1.04 (0.49, 2.22); *p* = 0.92	0.75 (0.42, 1.33); *p* = 0.33
MnBP (ng/ml)	All	1.31 (0.96, 1.78); *p* = 0.084	1.27 (0.83, 1.94); *p* = 0.27	0.98 (0.72, 1.32); *p* = 0.88
	Male	1.31 (0.83, 2.08); *p* = 0.24	1.32 (0.61, 2.86); *p* = 0.48	0.80 (0.48, 1.34); *p* = 0.40
	Female	1.35 (0.86, 2.13); *p* = 0.19	1.37 (0.80, 2.33); *p* = 0.25	1.09 (0.73, 1.63); *p* = 0.69
∑LMWP (ng/ml)	All	1.20 (0.92, 1.58); *p* = 0.19	1.12 (0.76, 1.64); *p* = 0.57	1.01 (0.80, 1.28); *p* = 0.92
	Male	1.03 (0.68, 1.57); *p* = 0.89	1.22 (0.62, 2.41); *p* = 0.56	1.08 (0.74, 1.57); *p* = 0.71
	Female	1.34 (0.92, 1.97); *p* = 0.13	1.07 (0.67, 1.73); *p* = 0.77	0.94 (0.68, 1.29); *p* = 0.70
MEP (ng/ml)	All	1.11 (0.87, 1.41); *p* = 0.40	1.00 (0.77, 1.42); *p* = 0.99	1.05 (0.85, 1.30); *p* = 0.63
	Male	0.91 (0.64, 1.29): *p* = 0.60	0.95 (0.55, 1.65); *p* = 0.86	1.28 (0.94, 1.74); *p* = 0.12
	Female	1.31 (0.93, 1.84); *p* = 0.12	1.00 (0.64, 1.55); *p* = 0.99	0.86 (0.63, 1.17); *p* = 0.33
MHBP (ng/ml)	All	0.76 (0.13, 4.49); *p* = 0.77	0.16 (0.01, 3.47); *p* = 0.24	0.68 (0.14, 3.24); *p* = 0.63
	Male	0.50 (0.03, 9.45); *p* = 0.65	0.39 (0.004, 37.36); *p* = 0.68	0.50 (0.04, 5.74); *p* = 0.58
	Female	1.77 (0.16, 20.21); *p* = 0.65	0.06 (0.001, 3.53); *p* = 0.17	0.97 (0.12, 7.75); *p* = 0.98
∑HMWP (ng/ml)	All	1.51 (0.75, 3.05); *p* = 0.25	1.71 (0.63, 4.66); *p* = 0.29	0.81 (0.44, 1.48); *p* = 0.49
	Male	1.68 (0.67,4.20); *p* = 0.27	1.30 (0.27, 6.22); *p* = 0.74	0.81 (0.35, 1.90); *p* = 0.63
	Female	1.33 (0.44, 3.99); *p* = 0.61	1.97 (0.49, 7.89); *p* = 0.34	0.77 (0.32, 1.86); *p* = 0.56
MBzP (ng/ml)	All	**1.78 (1.01, 3.14); *p* = 0.047**	1.51 (0.65, 3.52); *p* = 0.34	**0.27 (0.09, 0.83); *p* = 0.022**
	Male	**2.26 (1.14, 4.48); *p* = 0.019**	1.05 (0.18, 5.97); *p* = 0.96	**0.13 (0.02, 0.90); *p* = 0.038**
	Female	1.29 (0.37, 4.55); *p* = 0.69	2.06 (0.67, 6.35); *p* = 0.21	0.45 (0.11, 1.78); *p* = 0.26
MCPP (ng/ml)	All	1.02 (0.32, 3.24); *p* = 0.97	0.13 (0.01, 1.84); *p* = 0.13	1.12 (0.42, 2.98); *p* = 0.82
	Male	1.23 (0.26, 5.84); *p* = 0.80	0.49 (0.03, 7.81); *p* = 0.61	1.23 (0.34, 4.50); *p* = 0.75
	Female	0.85 (0.15, 4.79); *p* = 0.85	0.002 (0.0001, 2.15); *p* = 0.082	0.83 (0.18, 3.96); *p* = 0.82
MiDP (ng/ml)	All	1.04 (0.60, 1.82); *p* = 0.88	**2.18 (1.18, 4.01); *p* = 0.012**	0.93 (0.56, 1.53); *p* = 0.77
	Male	1.35 (0.49, 3.74); *p* = 0.57	**4.39 (1.16, 16.56); *p* = 0.029**	0.55 (0.15, 1.95); *p* = 0.35
	Female	0.99 (0.49, 2.00); *p* = 0.97	1.89 (0.88, 4.09); *p* = 0.10	1.25 (0.69, 2.24); *p* = 0.46
∑DEHP (ng/ml)	All	1.24 (0.65, 2.33); *p* = 0.51	0.70 (0.37, 1.84); *p* = 0.47	0.73 (0.40, 1.32); *p* = 0.29
	Male	1.58 (0.68, 3.65); *p* = 0.29	0.55 (0.12, 2.52); *p* = 0.44	0.81 (0.36, 1.82); *p* = 0.62
	Female	0.81 (0.28, 2.34); *p* = 0.70	0.81 (0.22, 2.94); *p* = 0.75	0.55 (0.22, 1.37); *p* = 0.20
MEHP (ng/ml)	All	1.21 (0.61, 2.42); *p* = 0.59	0.55 (0.19, 1.64); *p* = 0.29	0.73 (0.38, 1.39); *p* = 0.34
	Male	1.35 (0.54, 3.35); *p* = 0.53	0.31 (0.05, 1.87); *p* = 0.20	0.87 (0.37, 2.03); *p* = 0.75
	Female	1.01 (0.32, 3.13); *p* = 0.99	0.89 (0.22, 3.56); *p* = 0.87	0.52 (0.19, 1.40); *p* = 0.20
MECPP (ng/ml)	All	1.28 (0.59, 2.78); *p* = 0.53	0.87 (0.26, 2.78); *p* = 0.82	0.78 (0.37, 1.66); *p* = 0.52
	Male	1.39 (0.49, 3.93); *p* = 0.53	0.83 (0.13, 5.27); *p* = 0.85	0.54 (0.17, 1.80); *p* = 0.32
	Female	1.00 (0.29, 3.48); *p* = 0.99	0.77 (0.16, 3.77); *p* = 0.74	0.82 (0.28, 2.39); *p* = 0.72
∑DiNP (ng/ml)	All	1.30 (0.83, 2.03); *p* = 0.25	1.98 (0.96, 4.06); *p* = 0.063	1.11 (0.77, 1.60); *p* = 0.57
	Male	1.15 (0.64, 2.05); *p* = 0.64	1.89 (0.68, 5.27); *p* = 0.22	1.02 (0.61, 1.71); *p* = 0.94
	Female	1.68 (0.80, 3.55); *p* = 0.17	1.98 (0.70, 5.59); *p* = 0.20	1.16 (0.67, 2.00); *p* = 0.59
MiNP (ng/ml)	All	1.45 (0.83, 2.55); *p* = 0.19	**2.55 (1.08, 6.00); *p* = 0.032**	1.07 (0.67, 1.71); *p* = 0.77
	Male	1.25 (0.60, 2.57); *p* = 0.55	2.46 (0.74, 8.12); *p* = 0.14	0.96 (0.50, 1.82); *p* = 0.90
	Female	2.08 (0.80, 5.36); *p* = 0.13	2.67 (0.76, 9.80); *p* = 0.14	1.16 (0.57, 2.35); *p* = 0.68
MCiOP (ng/ml)	All	0.83 (0.26, 2.68); *p* = 0.76	0.19 (0.01, 2.62); *p* = 0.22	1.07 (0.43, 2.65); *p* = 0.89
	Male	0.95 (0.24, 3.85); *p* = 0.95	0.69 (0.05, 8.79); *p* = 0.77	1.54 (0.56, 4.24); *p* = 0.40
	Female	0.52 (0.06, 4.80); *p* = 0.57	0.06 (0.0001, 1.90); *p* = 0.081	0.33 (0.04, 2.67); *p* = 0.30

*Note*: Outcomes were modelled using multinomial logistic regression. Results were presented as relative risk ratios with no asthma ever (*n* = 701, 386 males and 315 females) as the reference group (Total observations *n* = 872, 484 males and 388 females). Models were adjusted for household income, maternal smoking, breastfeeding status and maternal age.

Abbreviations: MBzP, mono‐benzyl phthalate; MCiOP, mono‐carboxyiso‐nonyl phthalate; MCPP, mono‐3‐carboxypropyl phthalate; MECPP, mono‐(2‐ethyl‐5‐carboxypentyl) phthalate; MEP, mono‐ethyl phthalate; MEHP, Mono‐(2‐ethyl‐hexyl) phthalate; MHBP, mono‐(3‐hydroxybutyl) phthalate; MiBP, mono‐iso‐butyl phthalate; MiDP, mono‐iso‐decyl phthalate; MiNP, mono‐iso‐nonyl phthalate; MnBP, mono‐*n*‐butyl phthalate; ∑DEHP, the sum of di‐[2‐ethylhexyl] phthalate metabolites; ∑DiNP, the sum of di‐iso‐nonyl phthalate metabolites; ∑HMWP, molar sum of MBzP, MEHP, MECPP, MCPP, MiNP, MCiOP and MiDP high molecular weight phthalates; ∑LMWP, molar sum of low molecular weight phthalates MEP, MiBP, MnBP and MHBP; ∑MBP, sum of MiBP and MnBP.

**FIGURE 1:**
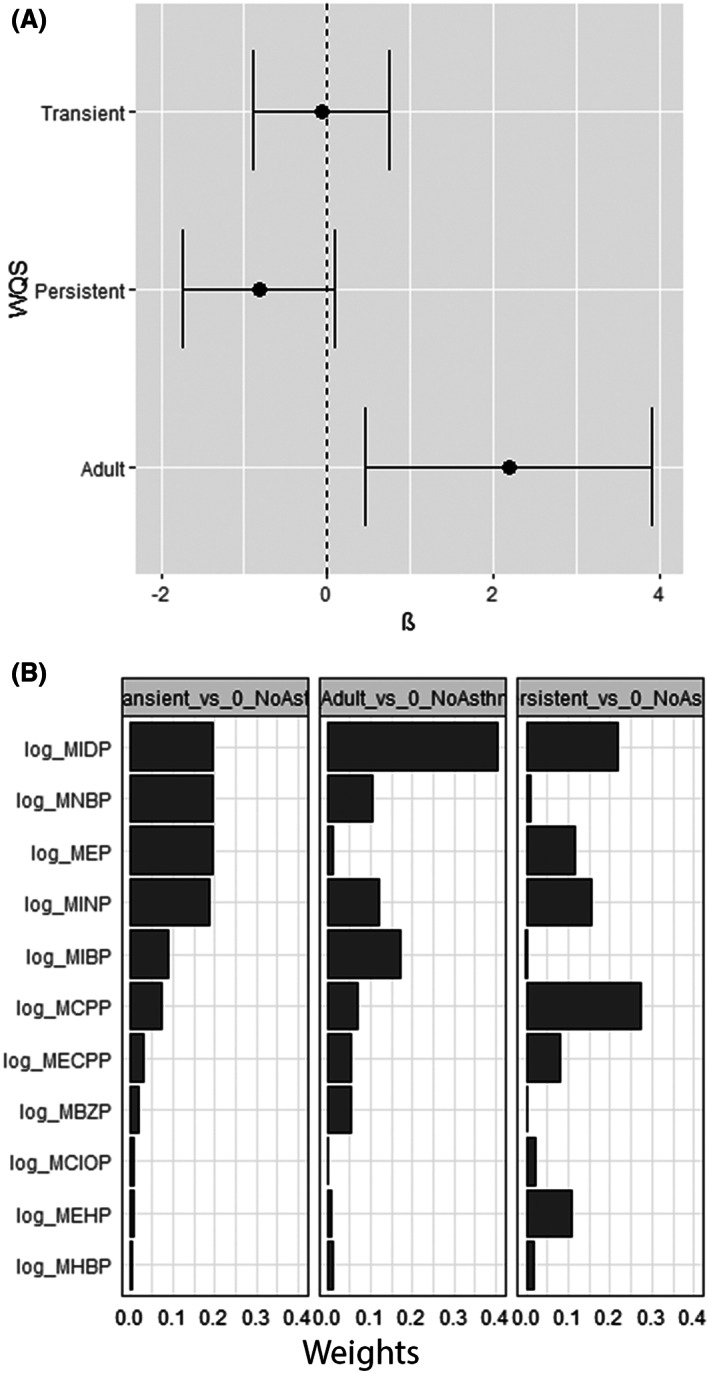
Coefficient estimates from a weighted quantile sum (WQS) regression of the WQS index of phthalate mixtures by asthma phenotype compared with no asthma as the reference group (A), and weights of all measured phthalates metabolites in association with asthma phenotypes (B). Models were adjusted for household income, maternal smoking, breastfeeding status and maternal age.

When examining the effects of phthalate mixtures on asthma phenotypes, the WQS index was significantly associated with a higher likelihood of adult asthma compared with no asthma (coefficient: 2.19, 95% CI: 0.46, 3.92; Figure 1A). Prenatal MiDP had the highest contributing weight of 40%, followed by MiBP at 17% (Figure 1B).

### The effects of prenatal BPA and phthalate exposure on lung function, and lung function trajectories from childhood to adulthood

Ten‐fold increases in prenatal ∑MBP (MiBP and MnBP) were associated with small but statistically significant higher FEV_1_ and FVC z‐scores in females, while increased levels of prenatal ∑LMWP (MEP) were also associated with significantly higher FEV_1_ and FVC z‐scores in females, and higher FEV_1_/FVC z‐scores in males. The only other significant finding for lung function in males was for MHBP, where increases were associated with 0.64 (95% CI: 0.08, 0.12) higher FEV_1_ z‐score. Effects of prenatal phthalate exposure on spirometry outcomes were also observed in females where 10‐fold increases in prenatal MBzP was associated with a 0.30 (95% CI: 0.08, 0.53) higher FEV_1_ z‐score, and MCPP associated with a 0.50 (95% CI: 0.10, 0.90) higher FEV_1_/FVC z‐score also in females only (Table S2 in the Supporting Information). MCiOP was the only phthalate where a statistically significant lower z‐score of 0.40 (95% CI: −0.79, −0.002) was found for FVC in females.

Prenatal exposure to MHBP, MCPP and MCiOP were significantly associated with decreased risk of being in low FEV_1_, FVC or the average‐low FEV_1_/FVC trajectories compared with the average trajectory. Ten‐fold increases in prenatal MHBP were associated with more than 80% decreased risk of being in the low FEV_1_, FVC or the average‐low FEV_1_/FVC trajectories when compared with the average trajectory in females. Prenatal MCPP exposure was associated with an 81% decreased risk of being in the average‐low FEV_1_/FVC trajectory compared to the average trajectory in females, while prenatal MCiOP was associated with a 69% decreased risk of being in the low FVC trajectory in males (Table 4). Sensitivity analyses with additional confounders did not alter results (Table S3 in the Supporting Information).

**TABLE 4:** Associations between log‐transformed BPA and phthalates with spirometry outcomes with lung function trajectory categories

		FEV_1_ (RRR [95% CI]; *p*‐value)		FVC (RRR [95% CI]; *p*‐value)		FEV_1_/FVC (RRR [95% CI]; *p*‐value)		
		Low (*n* = 270, 161 males and 109 females)	Above average (*n* = 34, 22 males and 12 females)	Low (*n* = 330, 200 males and 130 females	Above average (*n* = 46, 27 males and 19 females	Very low (*n* = 39, 25 males and 14 females)	Low‐average (*n* = 76, 36 males and 40 females)	Average‐low (*n* = 187, 119 males and 68 females)
BPA (μg/L)	All	0.98 (0.90, 1.07); *p* = 0.69	1.09 (0.90, 1.33); *p* = 0.38	1.04 (0.95, 1.14); *p* = 0.40	1.14 (0.95, 1.36); *p* = 0.16	1.04 (0.87, 1.23); *p* = 0.67	1.00 (0.88, 1.14); *p* = 0.99	1.04 (0.95, 1.14); *p* = 0.42
	Male	1.02 (0.92, 1.14); *p* = 0.66	1.11 (0.88, 1.39); *p* = 0.39	1.07 (0.96, 1.20); *p* = 0.23	1.10 (0.89, 1.37); *p* = 0.37	0.98 (0.81, 1.19); *p* = 0.87	0.92 (0.78, 1.09); *p* = 0.35	1.01 (0.90, 1.14); *p* = 0.85
	Female	0.89 (0.75, 1.04); *p* = 0.15	1.10 (0.72, 1.68); *p* = 0.65	0.98 (0.81, 1.15); *p* = 0.79	1.27 (0.86, 1.87); *p* = 0.24	1.27 (0.80, 2.01); *p* = 0.31	1.04 (0.84, 1.29); *p* = 0.70	1.11 (0.93, 1.33); *p* = 0.26
∑MBP (ng/ml)	All	0.83 (0.44, 1.04); *p* = 0.10	1.04 (0.68, 1.59); *p* = 0.85	0.99 (0.78, 1.26); *p* = 0.93	1.18 (0.79, 1.75); *p* = 0.42	0.73 (0.47, 1.12); *p* = 0.15	0.86 (0.62, 1.20); *p* = 0.39	0.88 (0.70, 1.13); *p* = 0.32
	Male	0.86 (0.61, 1.22); *p* = 0.39	0.75 (0.38, 1.47); *p* = 0.41	1.31 (0.89, 1.95); *p* = 0.17	1.27 (0.66, 2.47); *p* = 0.47	0.65 (0.32, 1.30); *p* = 0.22	1.15 (0.68, 1.96); *p* = 0.60	0.90 (0.62, 1.31); *p* = 0.60
	Female	0.80 (0.59, 1.09); *p* = 0.16	1.35 (0.72, 2.54); *p* = 0.35	0.82 (0.56, 1.14); *p* = 0.23	1.11 (0.65, 1.88); *p* = 0.70	0.70 (0.38, 1.31); *p* = 0.27	0.73 (0.48, 1.11); *p* = 0.14	0.87 (0.62, 1.22); *p* = 0.43
MiBP (ng/ml)	All	0.86 (0.65, 1.14); *p* = 0.31	0.99 (0.57, 1.72); *p* = 0.97	1.04 (0.76, 1.41); *p* = 0.82	1.19 (0.71, 1.98); *p* = 0.51	0.65 (0.37, 1.14); *p* = 0.14	1.11 (0.74, 1.68); *p* = 0.61	0.96 (0.71, 1.31); *p* = 0.80
	Male	0.91 (0.61, 1.37); *p* = 0.66	0.91 (0.43, 1.93); *p* = 0.80	1.45 (0.92, 2.29); *p* = 0.11	1.89 (0.89, 4.00); *p* = 0.097	0.70 (0.32, 1.54); *p* = 0.38	1.58 (0.85, 2.92); *p* = 0.15	0.92 (0.59, 0.41); *p* = 0.69
	Female	0.83 (0.56, 1.24); *p* = 0.36	1.01 (0.41, 2.50); *p* = 0.98	0.77 (0.49, 1.19); *p* = 0.24	0.77 (0.36, 1.66); *p* = 0.24	0.50 (0.20, 1.26); *p* = 0.14	0.87 (0.50, 1.52); *p* = 0.63	1.03 (0.65, 1.61); *p* = 0.91
MnBP (ng/ml)	All	0.81 (0.64, 1.03); *p* = 0.09	1.08 (0.70, 1.67); *p* = 0.73	1.00 (0.77, 1.29); *p* = 0.97	1.22 (0.81, 1.84); *p* = 0.34	0.69 (0.43, 1.12); *p* = 0.13	0.78 (0.54, 1.13); *p* = 0.19	0.86 (0.66, 1.11); *p* = 0.24
	Male	0.86 (0.59, 1.25); *p* = 0.43	0.74 (0.36, 1.53); *p* = 0.42	1.29 (0.85, 1.96); *p* = 0.24	1.12 (0.55, 2.30); *p* = 0.75	0.54 (0.24, 1.22); *p* = 0.14	1.02 (0.57, 1.82); *p* = 0.95	0.95 (0.64, 1.40); *p* = 0.80
	Female	0.77 (0.56, 1.08); *p* = 0.13	1.47 (0.79, 2.75); *p* = 0.22	0.84 (0.58, 1.20); *p* = 0.32	1.28 (0.75, 2.18); *p* = 0.37	0.73 (0.38, 1.41); *p* = 0.35	0.66 (0.41, 1.05); *p* = 0.077	0.79 (0.54, 1.14); *p* = 0.21
∑LMWP (ng/ml)	All	0.88 (0.73, 1.08); *p* = 0.22	1.11 (0.74, 1.65); *p* = 0.62	0.96 (0.78, 1.20); *p* = 0.74	1.19 (0.82, 1.72); *p* = 0.37	0.87 (0.60, 1.26); *p* = 0.46	0.91 (0.68, 1.23); *p* = 0.55	0.91 (0.73, 1.12); *p* = 0.37
	Male	0.81 (0.59, 1.12); *p* = 0.20	0.87 (0.49, 1.54); *p* = 0.63	1.12 (0.79, 1.57); *p* = 0.53	1.28 (0.72, 2.26); *p* = 0.40	0.58 (0.32, 1.07); *p* = 0.082	1.26 (0.76, 2.08); *p* = 0.37	0.86 (0.61, 1.21); *p* = 0.39
	Female	0.92 (0.71, 1.19); *p* = 0.51	1.30 (0.68, 2.44); *p* = 0.42	0.88 (0.66, 1.18); *p* = 0.39	1.10 (0.65, 1.83); *p* = 0.73	1.11 (0.64, 1.93); *p* = 0.77	0.82 (0.58, 1.18); *p* = 0.29	0.92 (0.69, 1.24); *p* = 0.60
MEP (ng/ml)	All	0.92 (0.78, 1.08); *p* = 0.30	1.03 (0.74, 1.44); *p* = 0.85	0.96 (0.80, 1.15); *p* = 0.63	1.13 (0.83, 1.54); *p* = 0.44	1.03 (0.75, 1.41); *p* = 0.86	1.05 (0.82, 1.34); *p* = 0.71	0.95 (0.79, 1.14); *p* = 0.58
	Male	0.88 (0.69, 1.12); *p* = 0.29	1.04 (0.67, 1.60); *p* = 0.86	1.99 (0.77, 1.29); *p* = 0.95	1.31 (0.84, 2.05); *p* = 0.24	0.75 (0.48, 1.17); *p* = 0.20	1.33 (0.90, 1.99); *p* = 0.15	0.91 (0.71, 1.18); *p* = 0.48
	Female	0.93 (0.73, 1.17); *p* = 0.53	0.91 (0.53, 1.57); *p* = 0.73	0.92 (0.71, 1.20); *p* = 0.54	0.95 (0.60, 1.49); *p* = 0.82	1.52 (0.92, 2.51); *p* = 0.10	0.97 (0.70, 1.34); *p* = 0.83	0.97 (0.74, 1.26); *p* = 0.80
MHBP (ng/ml)		**0.24 (0.08, 0.75); *p* = 0.014**	**5.56 (1.04, 29.88); *p* = 0.045**	**0.20 (0.06, 0.64); *p* = 0.007**	1.60 (0.30, 8.51); *p* = 0.58	0.41 (0.05, 3.28); *p* = 0.40	0.70 (0.15, 3.30); *p* = 0.65	0.38 (0.11, 1.28); *p* = 0.12
	Male	0.26 (0.04, 1.67); *p* = 0.16	4.46 (0.39, 51.49); *p* = 0.23	0.29 (0.05, 1.78); *p* = 0.18	0.92 (0.07, 12.87); *p* = 0.95	0.14 (0.002, 9.42); *p* = 0.36	0.95 (0.05, 17.66); *p* = 0.97	1.54 (0.26, 8.98); *p* = 0.63
	Female	**0.20 (0.04, 0.90); *p* = 0.036**	9.36 (0.63, 138.6); *p* = 0.10	**0.11 (0.02, 0.57); *p* = 0.008**	1.41 (0.14, 14.64); *p* = 0.77	0.44 (0.03, 6.57); *p* = 0.55	0.30 (0.04, 2.25); *p* = 0.24	**0.12 (0.02, 0.71); *p* = 0.019**
∑HMWP (ng/ml)	All	0.79 (0.49, 1.28); *p* = 0.34	1.42 (0.54, 3.77); *p* = 0.48	0.73 (0.43, 1.24); *p* = 0.25	1.18 (0.48, 2.91); *p* = 0.72	1.01 (0.42, 2.46); *p* = 0.98	0.91 (0.45, 1.85); *p* = 0.80	0.92 (0.55, 1.54); *p* = 0.76
	Male	0.84 (0.41, 1.70); *p* = 0.62	1.34 (0.36, 5.01); *p* = 0.67	0.66 (0.31, 1.41); *p* = 0.28	1.01 (0.26, 3.87); *p* = 0.99	1.24 (0.35, 4.36); *p* = 0.74	1.81 (0.54, 6.04); *p* = 0.33	1.42 (0.68, 2.97); *p* = 0.36
	Female	0.66 (0.33, 1.31); *p* = 0.23	2.00 (0.37, 10.7); *p* = 0.42	0.75 (0.35, 1.62); *p* = 0.47	1.39 (0.36, 5.30); *p* = 0.63	0.89 (0.21, 3.67); *p* = 0.87	0.67 (0.26, 1.71); *p* = 0.40	0.60 (0.28, 1.29); *p* = 0.19
MBzP (ng/ml)	All	0.62 (0.36, 1.07); *p* = 0.09	0.85 (0.32, 2.29); *p* = 0.76	1.08 (0.60, 1.94); *p* = 0.80	1.63 (0.73, 3.63); *p* = 0.23	0.63 (0.21, 1.89); *p* = 0.41	0.84 (0.38, 1.85); *p* = 0.67	0.82 (0.48, 1.42); *p* = 0.49
	Male	0.53 (0.23, 1.23); *p* = 0.14	0.38 (0.06, 2.39); *p* = 0.30	1.12 (0.52, 2.41); *p* = 0.76	1.56 (0.52, 4.71); *p* = 0.43	0.61 (0.12, 3.13); *p* = 0.55	1.17 (0.44, 3.15); *p* = 0.75	1.06 (0.52, 2.16); *p* = 0.88
	Female	0.73 (0.32, 1.63); *p* = 0.45	1.90 (0.47, 7.65); *p* = 0.36	1.00 (0.40, 2.51); *p* = 0.99	1.67 (0.44, 6.26); *p* = 0.45	0.47 (0.09, 2.52); *p* = 0.38	0.43 (0.13, 1.46); *p* = 0.18	0.65 (0.26, 1.62); *p* = 0.35
MCPP (ng/ml)	All	0.81 (0.40, 1.65); *p* = 0.57	1.48 (0.43, 5.13); *p* = 0.53	0.61 (0.29, 1.27); *p* = 0.19	0.55 (0.14, 2.17); *p* = 0.39	0.51 (0.12, 2.23); *p* = 0.38	0.62 (0.21, 1.84); *p* = 0.38	0.66 (0.31, 1.43); *p* = 0.29
	Male	0.66 (0.26, 1.67); *p* = 0.38	0.93 (0.19, 4.57); *p* = 0.93	0.43 (0.17, 1,12); *p* = 0.084	0.14 (0.02, 1.17); *p* = 0.07	0.47 (0.09, 2.52); *p* = 0.17	0.43 (0.13, 1.46); *p* = 0.24	0.65 (0.26, 1.62); *p* = 0.94
	Female	0.91 (0.29, 2.82); *p* = 0.87	1.44 (0.15, 14.10); *p* = 0.75	1.12 (0.31, 4.00); *p* = 0.87	1.58 (0.21, 11.98); *p* = 0.66	0.83 (0.11, 6.30); *p* = 0.86	0.71 (0.15, 3.27); *p* = 0.66	**0.19 (0.04, 0.86); *p* = 0.031**
MiDP (ng/ml)	All	1.17 (0.81, 1.71); *p* = 0.40	0.88 (0.39, 1.99); *p* = 0.77	1.42 (0.92, 2.20); *p* = 0.11	1.14 (0.55, 2.37); *p* = 0.72	1.02 (0.52, 1.97); *p* = 0.96	1.08 (0.63, 1.85); *p* = 0.77	0.94 (0.62, 1.41); *p* = 0.76
	Male	1.39 (0.59, 3.29); *p* = 0.46	1.76 (0.37, 8.31); *p* = 0.47	1.92 (0.68, 5.42); *p* = 0.22	2.36 (0.44, 12.80); *p* = 0.32	2.08 (0.53, 8.13); *p* = 0.29	1.79 (0.47, 6.76); *p* = 0.39	0.79 (0.31, 2.00); *p* = 0.62
	Female	1.13 (0.73, 1.77); *p* = 0.58	0.99 (0.33, 2.97); *p* = 0.99	1.27 (0.76, 2.12); *p* = 0.36	1.00 (0.41, 2.46); *p* = 0.99	0.97 (0.40, 2.35); *p* = 0.96	0.97 (0.51, 1.84); *p* = 0.92	1.04 (0.64, 1.69); *p* = 0.88
∑DEHP (ng/ml)	All	0.81 (0.52, 1.26); *p* = 0.35	1.24 (0.53, 2.90); *p* = 0.63	0.73 (0.45, 1.18); *p* = 0.20	1.06 (0.48, 2.37); *p* = 0.88	1.09 (0.48, 2.46); *p* = 0.83	0.89 (0.46, 1.71); *p* = 0.73	0.98 (0.61, 1.58); *p* = 0.96
	Male	0.91 (0.48, 1.71); *p* = 0.76	1.09 (0.34, 3.51); *p* = 0.88	0.74 (0.38, 1.46); *p* = 0.38	0.81 (0.24, 2.69); *p* = 0.73	1.11 (0.35, 3.48); *p* = 0.86	1.21 (0.42, 3.53); *p* = 0.72	1.13 (0.58, 2.21); *p* = 0.71
	Female	0.65 (0.34, 1.24); *p* = 0.19	1.66 (0.42, 6.64); *p* = 0.47	0.70 (0.34, 1.42); *p* = 0.32	1.47 (0.47, 4.60); *p* = 0.50	1.20 (0.35, 4.11); *p* = 0.77	0.90 (0.37, 2.15); *p* = 0.80	0.81 (0.39, 1.67); *p* = 0.57
MEHP (ng/ml)	All	0.83 (0.51, 1.35); *p* = 0.45	1.35 (0.52, 3.46); *p* = 0.54	0.85 (0.50, 1.45); *p* = 0.56	1.21 (0.49, 2.92); *p* = 0.68	0.68 (0.27, 1.72); *p* = 0.41	0.61 (0.29, 1.28); *p* = 0.19	1.04 (0.62, 1.77); *p* = 0.87
	Male	0.92 (0.46, 1.85); *p* = 0.83	1.03 (0.29, 3.72); *p* = 0.96	0.85 (0.41, 1.79); *p* = 0.68	0.79 (0.21, 2.99); *p* = 0.73	0.61 (0.16, 2.33); *p* = 0.47	0.82 (0.25, 2.67); *p* = 0.74	1.37 (0.67, 2.83); *p* = 0.39
	Female	0.69 (0.34, 1.40); *p* = 0.30	2.97 (0.61, 14.58), *p* = 0.18	0.82 (0.37, 1.82); *p* = 0.63	2.14 (0.59, 7.72); *p* = 0.25	0.90 (0.23, 3.56); *p* = 0.88	0.55 (0.20, 6.40); *p* = 0.24	0.74 (0.33, 1.66); *p* = 0.46
MECPP (ng/ml)	All	0.83 (0.49, 1.41); *p* = 0.50	0.73 (0.24, 2.20); *p* = 0.58	0.69 (0.39, 1.22); *p* = 0.20	0.77 (0.28, 2.07); *p* = 0.60	1.59 (0.62, 4.08); *p* = 0.34	1.46 (0.68, 3.16); *p* = 0.33	1.01 (0.56, 0.81); *p* = 0.97
	Male	0.77 (0.35, 1.68); *p* = 0.51	0.48 (0.09, 2.49); *p* = 0.38	0.60 (0.27, 1.36); *p* = 0.23	0.37 (0.07, 1.99); *p* = 0.25	1.68 (0.45, 6.36); *p* = 0.44	1.90 (0.57, 0.29); *p* = 0.30	0.95 (0.40, 2.24); *p* = 0.90
	Female	0.78 (0.36, 1.68); *p* = 0.52	0.60 (0.09, 3.97); *p* = 0.60	0.82 (0.34, 1.94); *p* = 0.65	1.21 (0.30, 4.98); *p* = 0.79	1.27 (0.27, 5.94); *p* = 0.76	1.79 (0.63, 5.12); *p* = 0.28	1.00 (0.40, 2.45); *p* = 0.99
∑DiNP (ng/ml)	All	0.92 (0.69, 1.21); *p* = 0.54	1.40 (0.74, 2.67); *p* = 0.30	0.83 (0.60, 1.14); *p* = 0.24	1.21 (0.67, 2.17); *p* = 0.53	1.16 (0.67, 2.00); *p* = 0.60	1.06 (0.69, 1.63); *p* = 0.79	1.00 (0.74, 1.36); *p* = 0.99
	Male	0.91 (0.61, 1.36); *p* = 0.64	1.29 (0.57, 2.93); *p* = 0.54	0.74 (0.47, 1.16); *p* = 0.19	1.25 (0.52, 2.98); *p* = 0.62	1.43 (0.66, 3.06); *p* = 0.36	1.61 (0.79, 3.29); *p* = 0.19	1.28 (0.83, 1.97); *p* = 0.26
	Female	0.89 (0.60, 1.32); *p* = 0.57	1.48 (0.47, 4.69); *p* = 0.51	0.93 (0.59, 1.46); *p* = 0.75	1.07 (0.46, 2.47); *p* = 0.88	0.78 (0.34, 1.82); *p* = 0.57	0.78 (0.45, 1.36); *p* = 0.38	0.78 (0.50, 1.22); *p* = 0.28
MiNP (ng/ml)	All	0.90 (0.63, 1.29); *p* = 0.58	1.59 (0.72, 3.50); *p* = 0.25	0.81 (0.54, 1.20); *p* = 0.29	1.28 (0.63, 2.63); *p* = 0.49	1.09 (0.55, 2.15); *p* = 0.81	1.06 (0.62, 1.84); *p* = 0.82	0.93 (0.63, 1.37); *p* = 0.71
	Male	0.92 (0.55, 1.52); *p* = 0.74	0.48 (0.55, 4.01); *p* = 0.44	0.76 (0.44, 1.31); *p* = 0.32	1.45 (0.51, 4.11); *p* = 0.48	1.40 (0.56, 3.52); *p* = 0.47	1.85 (0.77, 4.48); *p* = 0.17	1.28 (0.75, 2.18); *p* = 0.37
	Female	0.86 (0.51, 1.45); *p* = 0.57	1.66 (0.40, 6.90); *p* = 0.49	0.87 (0.48, 1.57); *p* = 0.64	0.98 (0.34, 2.81); *p* = 0.97	0.65 (0.22, 1.92); *p* = 0.43	0.66 (0.32, 1.36); *p* = 0.26	0.65 (0.36, 1.17); *p* = 0.15
MCiOP (ng/ml)	All	0.72 (0.34, 1.52); *p* = 0.39	0.73 (0.15, 3.55); *p* = 0.69	0.50 (0.23, 1.10); *p* = 0.09	0.48 (0.10, 2.20); *p* = 0.35	1.53 (0.39, 5.96); *p* = 0.54	1.24 (0.39, 3.94); *p* = 0.72	1.72 (0.74, 3.98); *p* = 0.21
	Male	0.60 (0.23, 1.55); *p* = 0.29	0.46 (0.05, 4.06); *p* = 0.49	**0.31 (0.11, 0.89); *p* = 0.029**	0.15 (0.01, 2.16); *p* = 0.16	1.43 (0.20, 10.07); *p* = 0.72	1.13 (0.18, 7.24); *p* = 0.90	2.38 (0.81, 6.98); *p* = 0.11
	Female	0.78 (0.20, 3.01); *p* = 0.72	0.90 (0.04, 19.01); *p* = 0.95	1.53 (0.31, 7.61); *p* = 0.60	2.28 (0.22, 23.66); *p* = 0.49	1.76 (0.18, 17.23); *p* = 0.63	1.87 (0.33, 10.66); *p* = 0.48	0.58 (0.11, 3.19); *p* = 0.53

*Note*: Outcomes were modelled using multinomial logistic regression. Results were presented as relative risk ratios with the average trajectory (FEV_1_: *n* = 223, 137 male and 86 females; FVC: *n* = 151, 93 males and 58 females; FEV_1_/FVC: *n* = 225 males, 140 males and 85 females) as the reference group. Models adjusted for household income, maternal smoking, breastfeeding status and maternal age.

Abbreviations: MBzP, mono‐benzyl phthalate; MCiOP, mono‐carboxyiso‐nonyl phthalate; MCPP, mono‐3‐carboxypropyl phthalate; MECPP, mono‐(2‐ethyl‐5‐carboxypentyl) phthalate; MEHP, mono‐(2‐ethyl‐hexyl) phthalate; MEP, mono‐ethyl phthalate; MHBP, mono‐(3‐hydroxybutyl) phthalate; MiBP, mono‐iso‐butyl phthalate; MiDP, mono‐iso‐decyl phthalate; MiNP, mono‐iso‐nonyl phthalate; MnBP, mono‐*n*‐butyl phthalate; ∑DEHP, the sum of di‐[2‐ethylhexyl] phthalate metabolites; ∑DiNP, the sum of di‐iso‐nonyl phthalate metabolites; ∑HMWP, molar sum of MBzP, MEHP, MECPP, MCPP, MiNP, MCiOP and MiDP high molecular weight phthalates; ∑LMWP, molar sum of low molecular weight phthalates MEP, MiBP, MnBP and MHBP; ∑MBP, sum of MiBP and MnBP.

**FIGURE 2:**
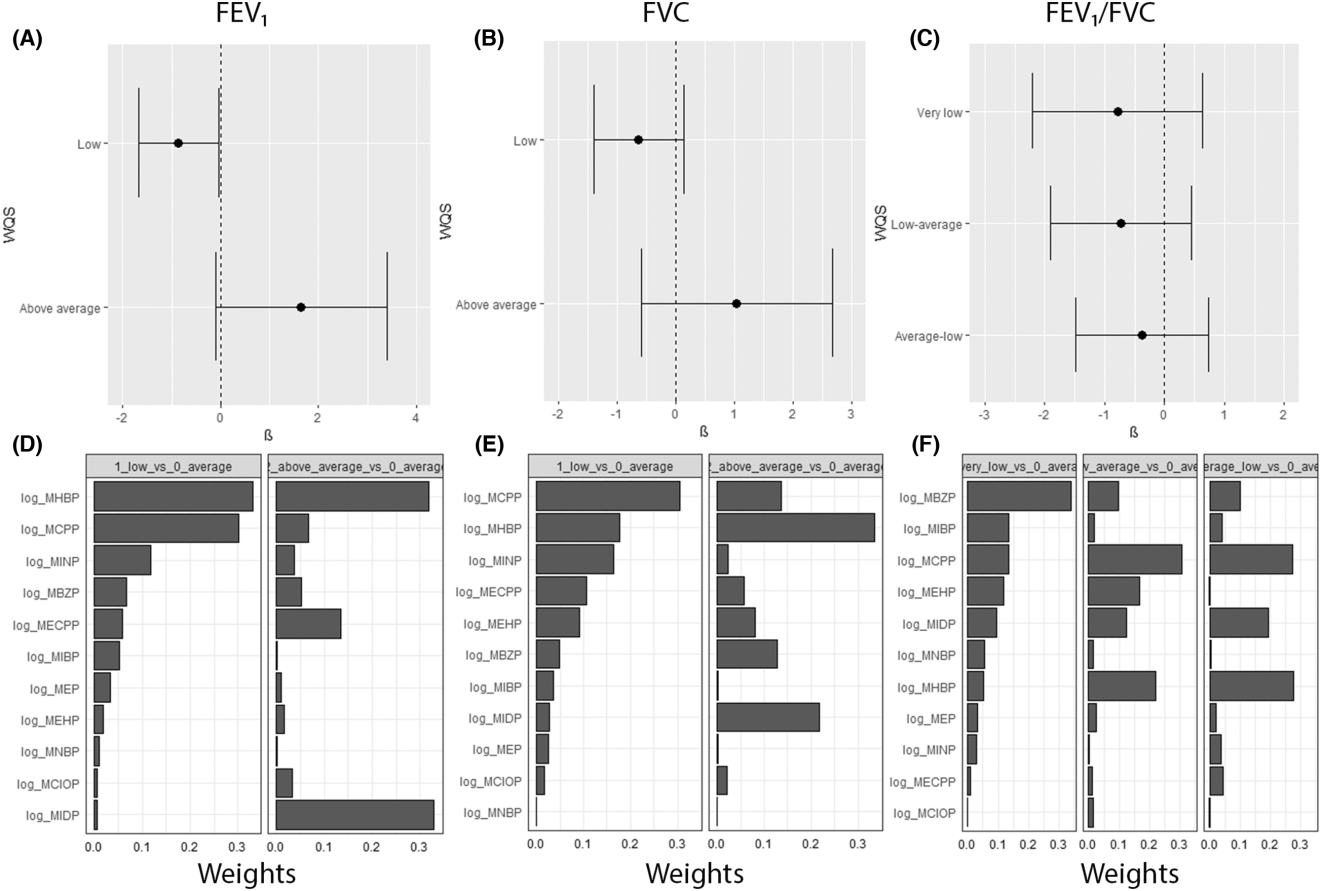
Coefficient estimates from a weighted quantile sum (WQS) regression of the WQS index of phthalate mixtures for FEV_1_ (A), FVC (B) and FEV_1_/FVC (C) trajectories, and weights of all measured phthalates metabolites in association with FEV_1_ (D), FVC (E) and FEV_1_/FVC (F) trajectories. All comparisons were made with the average trajectory as the reference group. Models were adjusted for household income, maternal smoking, breastfeeding status and maternal age.

WQS regression models showed that the WQS index was significantly associated with lower likelihood of belonging to the low FEV_1_ trajectory compared with average (coefficient: −0.86, 95% CI: −1.68, −0.05; Figure 2A), with MHBP and MCPP contributing the highest weights (Figure 2D). The WQS index was not significantly associated with other spirometry outcomes.

## DISCUSSION

This study shows that prenatal exposure to plastic by‐products, BPA and phthalates and their metabolites, are associated with increased odds for asthma and altered lung function from childhood to adulthood. Prenatal BPA and phthalates were associated with increased odds for asthma, as well as increased risks for persistent and adult asthma, respectively. Prenatal phthalate exposure, but not BPA was significantly associated with improved lung function, as well as altered risks of being in different lung function trajectories. These findings suggest that prenatal exposure to plastic by‐products may have long‐term effects on the lung health of offspring, and that these effects may be modified by sex of the offspring.

Two previous studies have shown an association between prenatal BPA exposure with increased asthma risk in boys,^20^, ^21^ while another found no differences for asthma regardless of sex.^5^ In our study, we found an association between prenatal BPA levels with increased odds for asthma throughout life in males, but not females. Prenatal BPA exposure was also associated with increased risk for persistent asthma in males. Despite associations with asthma, no associations were observed for prenatal BPA exposure with lung function outcomes. Spanier et al. showed associations between prenatal BPA exposure with decreased lung function at 4 years, which was no longer present at 5 years.^22^ While this may be due to short‐term variability in participants, this suggests airway dysfunction may be present at younger ages but is alleviated at later timepoints. There are no studies that have reported associations with lung function outcomes at older ages. The effects observed in our study in males may be due to immunomodulatory factors, and not due to changes in lung structure, as we did not find any effects of BPA on lung function. BPA is an endocrine disrupter with estrogenic properties, binding to oestrogen receptors,^23^ which may promote the polarization of CD4+ T cells to a Type 2T helper cell response associated with allergic inflammation.^24^ The increased binding of BPA to oestrogen receptors on immunomodulatory cells in males may in part explain the sex‐specific effects of BPA on asthma. Moreover, we did not find associations with allergy. Allergy is a strong risk factor for persistent asthma,^25^ which may contribute to findings of no significant associations with persistent asthma.

Current evidence for the role of prenatal phthalate exposure in allergic disease is conflicting, with previous studies solely investigating ∑DEHP reporting increased,^5^ decreased,^26^ and no associations^4^ with risk of asthma. In our study, we did not observe associations between prenatal ∑DEHP exposure with any of the respiratory outcomes reported. We found that prenatal ∑DiNP, including MiNP, exposure increased the odds of asthma in females. MiBP and MiDP was associated with increased risk for an adult asthma phenotype in males and this was further supported by the mixture model analysis, while MBzP with a higher risk for transient asthma but lower risk for persistent asthma in males. Two studies have also found that prenatal MBzP exposure was associated with increased odds of asthma in children,^5^, ^26^ supporting in part our findings of a higher risk for transient asthma in males.

Despite most published studies suggesting phthalates are associated with impaired lung function, the results of this study revealed that prenatal phthalate exposure was mostly associated with improved spirometry outcomes. Two studies examined prenatal phthalate exposure on lung function in children where the first found MCiOP and MBzP was associated with lower FEV_1_ percent predicted in children aged 7 years.^4^ The second study only included boys in their cohort and found MiBP and ∑DEHP also associated with lower FEV_1_ percent predicted at age 5 years. It is unclear why we found improved lung function, but no associations with better asthma outcomes, it is possible that postnatal exposures that were not accounted for independently influenced these outcomes.

Potential limitations of this study were that BPA and phthalate measurements were conducted in maternal serum samples instead of traditional measurements in urine. We are therefore unable to directly determine if the exposure levels in this study is comparable to other birth cohort studies. Pilot studies were conducted to measure first‐step and second‐step metabolites, which are thought to reflect true exposure and confirmed sample and analyses robustness.^13^ Nonetheless, while there are limitations to measurements in serum, urinary BPA and phthalate concentrations may also be influenced by urinary dilution, kidney function and metabolic detoxification pathways. A further limitation is that individual postnatal exposure to BPA and phthalates also have effects on lung function and asthma.^27^, ^28^ As only prenatal BPA and phthalates were measured in this article, we were not able to control for postnatal exposures that may have influenced the results reported.

To our knowledge, this is the first study to examine the associations between prenatal exposure to plastic by‐products, BPA and phthalates on longitudinal outcomes in children into adulthood and show that exposure may have long‐term effects on respiratory health. Our findings support previous reports that the effects of these compounds on respiratory health outcomes are modified by sex. To date, most studies studying the effects of prenatal exposure to BPA and phthalates have focused on childhood. Further investigation in other longitudinal cohort studies is warranted to confirm our findings of long‐term effects following early‐life exposure.

## AUTHOR CONTRIBUTION

**Rachel E. Foong:** Conceptualization (lead); formal analysis (lead); investigation (lead); methodology (lead); project administration (lead); writing – original draft (lead). **Peter Franklin:** Conceptualization (equal); supervision (supporting); writing – review and editing (equal). **Francesca Sanna:** Formal analysis (supporting); writing – review and editing (supporting). **Graham L. Hall:** Conceptualization (equal); investigation (supporting); project administration (supporting); supervision (supporting); writing – review and editing (equal). **Peter D. Sly:** Data curation (equal); writing – review and editing (supporting). **Eric B. Thorstensen:** Data curation (supporting); writing – review and editing (supporting). **Dorota A. Doherty:** Formal analysis (supporting); supervision (supporting); writing – review and editing (supporting). **Jeffrey A. Keelan:** Data curation (supporting); investigation (supporting); writing – review and editing (supporting). **Roger J. Hart:** Conceptualization (equal); data curation (equal); writing – review and editing (equal).

## CONFLICT OF INTEREST

None declared.

## HUMAN ETHICS APPROVAL DECLARATION

This study was approved by the Curtin University Human Research Ethics Committee (HRE2020‐0335), with each follow‐up receiving separate research ethics approval. All participants provided written informed consent. At the 5 and 13‐year follow‐up, this was provided by the primary caregiver.

## Supporting information


Supporting Information S1

**Visual Abstract** Longitudinal effects of prenatal exposure to plastic‐derived chemicals and their metabolites on asthma and lung function from childhood into adulthood

## Data Availability

The data that support the findings of this study are available from the corresponding author upon reasonable request. The data are not publicly available due to privacy or ethical restrictions.
