# Supplementary material for: Longitudinal effects of prenatal exposure to plastic‐derived chemicals and their metabolites on asthma and lung function from childhood into adulthood
Source: Respirology. 2022 Oct 2;28(3):236–46. doi: 10.1111/resp.14386 (PMC10946907; doi:10.1111/resp.14386)
Supplement: Supplementary file 1 — Supporting Information S1 [file RESP-28-236-s001.docx]

**SUPPORTING INFORMATION**

**Longitudinal effects of prenatal exposure to plastic-derived chemicals and their metabolites on asthma and lung function from childhood into adulthood**

Rachel E Foong^1,2^, Peter Franklin^3^, Francesca Sanna^1^, Graham L Hall^1,2^, Peter D Sly^4^, Eric B Thorstensen^5^, Dorota A Doherty^6,8^, Jeffrey A Keelan^6,7^, Roger J Hart^6,8^

*^1^Wal-Yan Centre for Respiratory Research, Telethon Kids Institute, Perth, Western Australia*

*^2^School of Allied Health, Curtin University, Perth, Western Australia*

*^3^School of Population Health, University of Western Australia, Perth, Western Australia*

*^4^Child Health Research Centre, The University of Queensland, South Brisbane, Queensland*

*^5^Liggins Institute, University of Auckland, New Zealand*

*^6^Women and Infants Research Foundation, King Edward Memorial Hospital, Perth Western Australia*

*^7^School of Biomedical Sciences, University of Western Australia, Perth, Western Australia*

*^8^Division of Obstetrics and Gynaecology, University of Western Australia, Perth, Western Australia*

**Correspondence:**

Rachel E Foong

Email: [Rachel.Foong@telethonkids.org.au](mailto:Rachel.Foong@telethonkids.org.au)

**Appendix S1-METHODS & RESULTS**

**Methods**

*Study population*

The Raine Study is a longitudinal birth cohort where from 1989-1991 2834 women between 16 to 18 weeks pregnancy were recruited into the study at King Edward Memorial Maternity Hospital, the sole tertiary obstetric referral hospital in Western Australia, and surrounding private clinics in Perth, initially in a randomized control trial to determine the effects of repeated ultrasound imaging during pregnancy.^1^ Liveborn offspring of consenting parents were then enrolled in a longitudinal cohort study with the objective of determining how events during pregnancy and childhood influence health in later life. The current study took advantage of bisphenol A (BPA) and phthalate previously measured in maternal serum samples, of which 1377 samples were available from Raine Study participants that were cryobanked at enrolment for BPA and phthalate measurement.

*BPA and phthalate metabolite measurement*

Pooled maternal serum samples were spiked with an internal BPA standard (BPA d16; Cambridge Isotope Laboratory, Woburn, MA, USA) and incubated with glucuronidase (255 U)/sulphatase (22.5 U) at pH 5 at 37°C for 3 hours. BPA was extracted in glass tubes with ethyl acetate and lyophilized by vacuum concentration then redissolved in 70 µL of mobile phase (methanol:water at a ratio of 80:20). Separation was achieved via HPLC using a Phenomenex Luna C18 HST 2.5 µm 100 × 3.0 mm column at 40°C (Phenomenex, Torrance, CA) under isocratic conditions. Mass spectrometry was performed using a Finnigan TSQ QuantumUltra AM triple quadrupole mass spectrometer (Thermo Electron Corp., San Jose, CA) in APCI negative ion mode. All reagents were filtered through Empore RP-SDB extraction discs (Phenomenex) to prevent any potential BPA contamination. Glass tubes and glass HPLC inserts were used, and all plastic tips and disposables were BPA-free. Blanks were included throughout the deconjugation, extraction and analysis steps to exclude external contamination; BPA levels were below the limit of detection (LOD) of 0.005 µg/L in blanks.

Eleven phthalate metabolites: mono-ethyl phthalate (MEP), mono-n-butyl phthalate (MnBP), mono-iso-butyl phthalate (MiBP), mono-benzyl phthalate (MBzP), mono-n-pentyl phthalate (MPP), mono-(2-ethyl-hexyl) phthalate (MEHP), mono(2-ethyl-5-carboxypentyl) phthalate (MECPP), mono-octyl phthalate (MOP), mono-iso-nonyl phthalate (MiNP), mono-carboxyiso-nonyl phthalate (MCiOP) and mono-iso-decyl phthalate (MiDP) were measured by isotope-diluted liquid chromatography–tandem mass spectrometry (LC–MS/MS), after deconjugation by enzymatic hydrolysis and purification by solid phase extraction. Standards for MEP, MnBP, MBzP, MEHP, MiNP (Cambridge Isotope Laboratory, Woburn, MA, USA), MiBP, MECPP, MCiOP (IDM, Teltow, Germany) were prepared by dilution with 50% acetonitrile to a concentration of 10 μg/mL. Samples were purified by automated solid-phase extraction with the analytes eluted with 1.5 mL pure acetonitrile followed by 1.5 mL ethyl acetate. The eluates were then evaporated to dryness (45°C, 5–15 psi N2), and the residues of total metabolites resuspended in 200 μL of resuspension solution (1% formic acid in 60% acetonitrile/water). High-performance liquid chromatograph (HPLC) (Surveyor, ThermoFinnigan, San Jose, CA) coupled with a tandem MS (Finnigan TSQ Quantum Ultra triple-quadrupole MS, ThermoElectron, San Jose, CA) in combination with the Xcalibur software (ThermoFinnigan, Bellefonte, PA) was used for detection and quantification of analytes. MEHP and MECPP were combined and expressed as the sum of DEHP metabolites (∑DEHP) and MiNP and MCiOP were combined and expressed as the sum of di-iso-nonyl phthalate (DiNP) metabolites (∑DiNP). To combine the metabolites, the sum of each metabolite expressed in molar concentration was multiplied with their respective parent molecular weight.

Allergic sensitization was assessed in participants by skin prick testing (SPTs). At the 5-year follow-up, allergens tested included cat dander, rye grass pollen, house dust mite, egg white and mould. At 13 and 22 years, allergens included cat dander, rye grass pollen, house dust mite, mould, peanut, grass mix and couch grass. Results of SPT responses to any of these allergens were used to generate the variables for allergic sensitization at 5, 13 and 22 years where a recorded wheal size of more than 3mm was defined as a positive response.

Spirometry was conducted at the 5, 13 and 22-year follow-ups in the Raine Study according to the American Thoracic Society in place at each time point ^2^. FEV_1_ and FVC were measured from at least three acceptable and repeatable forced expiratory curves. The largest FEV_1_ and FVC were recorded after examining the data from all acceptable curves within the defined repeatability limits. FEV_1_/FVC was calculated as the ratio of forced expiratory volume in the first one second to the forced vital capacity. Reference values were derived from the Global Lung Function Initiative (GLI) reference equations ^3^ with FEV_1_, FVC and FEV_1_/FVC reported as outcome measures. Using the GLI predicted equations, the raw FEV_1_, FVC and FEV_1_/FVC outcomes were converted to z-scores and standardized at all age groups and has been validated in an Australasian population ^4^.

Potential confounders were identified based on the literature and a direct acyclic graph (DAG) was created using DAGitty.^5^ DAGs are qualitative causal path diagrams used to determine covariate adjustment sets to remove structural confounding bias. By drawing pathways between co-variates, testable restrictions can be identified where there are no direct causal paths between pairs of variables. the minimal sufficient adjustment sets for estimating the total effect of BPA and phthalates on respiratory outcomes were identified using a DAG (Supplementary material Figure S2). These included: i) quartiles of predicted equivalised total disposable household income or reported household income; ii) maternal smoking during pregnancy (yes/no); iii) maternal age at birth; and iv) ever breastfed (yes/no). Information on covariates were obtained by questionnaire. Additional potential confounders not included in the minimal sufficient adjustment set were maternal asthma and weight-for-age z-scores at birth using World Health Organization child growth standards.^6^ Sensitivity analyses were performed including these additional co-variates.

*Statistical methods*

All BPA and phthalate values < limit of detection (LOD) were replaced by the LOD divided by $\sqrt{2}$ for statistical analysis. As the distribution of the exposure data were skewed, the data were log-transformed. Random effects logistic regression models were used to examine the associations between BPA and phthalates with repeated binary asthma and allergy outcomes at 5, 13 and 22 years. For phthalates exposure variables, the sum of MiBP and MnBP (∑MBP), ∑DEHP, ∑DiNP, molar sum of low molecular weight phthalates MEP, MiBP, MnBP and MHBP (∑LMWP), molar sum of MBzP, MEHP, MCMHP, MECPP, MCPP, MiNP, MCiOP and MiDP high molecular weight phthalates (∑HMWP) were also used together with the individual phthalates. The correlation between the exposures were plotted. Linear mixed-effects models with random intercepts for individual participants were used to determine effects of prenatal exposures with repeated spirometry outcomes. Lung function trajectories were plotted ^7^ using group-based trajectory modelling.

Trajectories for FEV1, FVC and FEV1/FVC (z-scores) were modeled used group-based trajectory modelling. Semi-parametric mixture models were estimated using “traj” plugin ^8^ from STATA (version SE 16.1; StataCorp, College Station, TX). Given the nature of FEV1 and FVC values (z-scores), a censored normal model was fitted. Group based trajectory modelling identified four distinct lung function trajectories of FEV_1_, FVC and FEV_1_/FVC that best fitted our data with a membership probability of >0.7 for each group. FEV_1_ and FVC were characterized by four trajectories labelled respectively: “very low”, “low”, “average” and “above average”. Based on their shape, lung function trajectories of FEV_1_/FVC were labelled as “very low”, “low-high”, “high-low” and “average” (Figure S2).

For the purposes of the current analyses due to the small proportion of participants in the “very low” trajectories (less than 5%), the “very low” and “low” trajectories for FEV_1_ and FVC were merged to allow for sufficient power to undertake multinomial logistic regression analyses, where a minimum of ten events per variable is recommended ^9^. Multinomial logistic regression was used for associations between prenatal exposures and asthma phenotypes and lung function trajectory groups and the estimated coefficients transformed to relative-risk ratios. Analyses were also stratified by sex. Weighted quantile sum (WQS) regression model was used to evaluate the effects of mixed exposure to the 11 phthalate metabolites, in conjunction with multinomial logistic regression.^10^ WQS regression examines chemical exposures based on the weights empirically determined by bootstrap sampling, thus better representing real-life complex exposures. The weighted mean of the bootstrap weights is applied to phthalate concentration quantiles and summed to create an index of the combined exposure, the weighted quantile sum. The exposure index is then used to assess the association between the WQS index and the outcome of interest using the hold-out validation subset of the data with the following model for multinomial outcomes:

$$\mathrm{logit} \pi_{j}=\log(\frac{\pi_{j}}{\pi_{1}})=\beta_{0}+\beta_{1}(\sum_{i=1}^{c} w_{i}q_{i})+z^{'}\varphi$$

Where $\beta_{0}$ is the intercept, $z^{'}$ a vector of covariates, with the sum term the index for *c* items where $w_{i}$ is the weights for each exposure and $q_{i}$ denotes the quantiles for each *i*th phthalate metabolites, and $\beta_{1}$ the regression coefficient for the weight index.^10^

In fitting the generalized WQS model, we used four quantiles with 100 bootstraps and a 60–40 split of training and validation datasets. We reported the results as ORs with 95% confidence intervals. We also assessed the important chemical exposures in the group using the estimated weights. Models were adjusted for potential confounding. Hypothesis tests were two sided with p-values of <0.05 considered statistically significant. In this exploratory, hypothesis-generating study of associations between maternal BPA phthalates and offspring’s respiratory outcome data, no adjustments for multiple testing were made, as recommended by the American Statistical Association for such exploratory analyses.^11^ Analyses were conducted using Stata SE 16.1 (College Station, TX, USA) or in R (Version 3.1.4) (Vienna, Austria).

**Results**

*Sensitivity analyses*

Inclusion of additional confounders maternal asthma and weight-for-age z-scores at birth did not result in changes to overall findings for the associations between prenatal BPA and phthalate metabolites with asthma phenotypes apart from prenatal ∑DiNP now demonstrating a statistically significant association with increased odds for adult asthma compared with no asthma (RRR: 2.14, 95% CI: 0.96, 4.06) (Table S2).

Similarly for lung function trajectories, the only statistically significant change was for prenatal MHBP exposure, where there was an increased likelihood of belonging in the above average trajectory compared with the average trajectory in females (RRR:19.15; 95% CI: 1.06, 348.46) (Table S3).

*Table S1. Associations between log-transformed bisphenol A (BPA) and phthalates‡ with asthma phenotypes with additional confounders for sensitivity analysis.*

|  | Sex | Transient Asthma  (n=66, 40 males and 26 females) | Adult Asthma  (n=26, 14 males and 12 females) | Persistent Asthma  (n=79, 44 males and 35 females) |
| --- | --- | --- | --- | --- |
|  |  | Relative Risk Ratios (95% Confidence Interval); p-value | | |
| BPA (µg/L) | All | **1.18 (1.00, 1.39); p=0.042** | 0.97 (0.81, 1.17); p=0.76 | **1.20 (1.03, 1.39), p=0.019** |
|  | Male | 1.24 (1.01, 1.54); p=0.042 | 1.22 (0.87, 1.72); p=0.24 | **1.23 (1.02, 1.49); p=0.032** |
|  | Female | 1.15 (0.87, 1.51); p=0.32 | 0.82 (0.64, 1.04); p=0.097 | 1.21 (0.93, 1.58); p=0.15 |
| ∑MBP (ng/mL) | All | 1.31 (0.97, 1.78); p=0.078 | 1.31 (0.88, 1.97);p=0.19 | 0.92 (0.69, 1.24); p=0.60 |
|  | Male | 1.22 (0.78, 1.92); p=0.39 | 1.72 (0.83, 3.59); p=0.15 | 0.79 (0.50, 1.25); p=0.31 |
|  | Female | 1.38 (0.90, 2.11); p=0.14 | 1.25 (0.73, 2.14); p=0.42 | 1.00 (0.89, 1.43); p=0.99 |
| MiBP (ng/ml) | All | 1.03 (0.78, 1.75); p=0.44 | 1.43 (0.86, 2.37); p=0.17 | 0.85 (0.57, 1.25); p=0.41 |
|  | Male | 1.03 (0.57, 1.87); p=0.92 | **2.56 (1.13, 5.80); p=0.024** | 0.84 (0.48, 1.47); p=0.53 |
|  | Female | 1.20 (0.68, 2.12); p=0.53 | 0.99 (0.47, 2.12); p=0.99 | 0.78 (0.44, 1.39); p=0.41 |
| MnBP (ng/mL) | All | 1.33 (0.97, 1.82); p=0.077 | 1.28 (0.84, 1.96); p=0.25 | 0.99 (0.72, 1.35); p=0.94 |
|  | Male | 1.27 (0.80, 2.02); p=0.31 | 1.44 (0.65, 3.22); p=0.37 | 0.81 (0.49, 1.33); p=0.40 |
|  | Female | 1.38 (0.88, 2.19); p=0.16 | 1.35 (0.78, 2.34); p=0.28 | 1.12 (0.74, 1.71); p=0.59 |
| ∑LMWP (ng/mL) | All | 1.22 (0.92, 1.62); p=0.16 | 1.12 (0.77, 1.63); p=0.55 | 1.04 (0.82, 1.33); p=0.76 |
|  | Male | 1.04 (0.68, 1.61); p=0.85 | 1.28 (0.62, 2.62); p=0.50 | 1.09 (0.75, 1.61); p=0.64 |
|  | Female | 1.33 (0.91, 1.93); p=0.14 | 1.06 (0.66, 1.70); p=0.81 | 0.97 (0.70, 1.34); p=0.84 |
| MEP (ng/mL) | All | 1.13 (0.88, 1.44); p=0.35 | 1.01 (0.72, 1.42); p=0.96 | 1.08 (0.87, 1.35); p=0.48 |
|  | Male | 0.94 (0.66, 1.35): p=0.74 | 0.93 (0.52, 1.66); p=0.80 | 1.32 (0.96, 1.81); p=0.087 |
|  | Female | 1.28 (0.92, 1.79); p=0.13 | 1.00 (0.65, 1.54); p=0.99 | 0.87 (0.63, 1.20); p=0.40 |
| MHBP (ng/mL) | All | 0.77 (0.13, 4.51); p=0.77 | 0.14 (0.01, 3.47); p=0.22 | 0.75 (0.16, 3.53); p=0.71 |
|  | Male | 0.43 (0.02, 7.87); p=0.57 | 0.30 (0.002, 42.40); p=0.63 | 0.47 (0.04, 5.31); p=0.55 |
|  | Female | 1.86 (0.16, 21.82); p=0.62 | 0.06 (0.001, 3.61); p=0.18 | 1.15 (0.14, 9.35); p=0.89 |
| ∑HMWP (ng/mL) | All | 1.57 (0.77, 3.18); p=0.22 | 1.84 (0.67, 5.08); p=0.24 | 0.83 (0.45, 1.54); p=0.55 |
|  | Male | 1.59 (0.67,4.20); p=0.32 | 1.55 (0.31, 7.81); p=0.60 | 0.79 (0.33, 1.88); p=0.60 |
|  | Female | 1.46 (0.47, 4.55); p=0.51 | 2.26 (0.55, 9.33); p=0.26 | 0.80 (0.32, 2.00); p=0.64 |
| MBzP (ng/mL) | All | **1.80 (1.01, 3.21); p=0.044** | 1.53 (0.64, 3.64); p=0.34 | **0.24 (0.07, 0.76); p=0.016** |
|  | Male | **2.08 (1.04, 4.49); p=0.038** | 1.10 (0.19, 6.28); p=0.92 | **0.12 (0.02, 0.84); p=0.032** |
|  | Female | 1.28 (0.38, 4.38); p=0.69 | 2.15 (0.66, 6.96); p=0.20 | 0.40 (0.09, 1.73); p=0.22 |
| MCPP (ng/mL) | All | 1.17 (0.35, 3.88); p=0.80 | 0.12 (0.01, 1.87); p=0.13 | 1.33 (0.48, 3.69); p=0.58 |
|  | Male | 1.24 (0.23, 6.73); p=0.80 | 0.66 (0.03, 13.18); p=0.79 | 1.27 (0.33, 4.99); p=0.73 |
|  | Female | 0.97 (0.16, 4.95); p=0.93 | 0.002 (0.0001, 1.96); p=0.077 | 1.09 (0.22, 5.33); p=0.92 |
| MiDP (ng/mL) | All | 1.05 (0.60, 1.84); p=0.85 | **2.19 (1.13, 3.90); p=0.019** | 0.85 (0.50, 1.43); p=0.54 |
|  | Male | 1.25 (0.45, 3.47); p=0.67 | **4.66 (1.18, 18.38); p=0.028** | 0.53 (0.15, 1.91); p=0.33 |
|  | Female | 0.97 (0.47, 1.96); p=0.93 | 1.91 (0.88, 4.18); p=0.10 | 1.10 (0.59, 2.05); p=0.75 |
| ∑DEHP (ng/mL) | All | 1.25 (0.66, 2.37); p=0.49 | 0.73 (0.27, 1.95); p=0.53 | 0.78 (0.43, 1.42); p=0.42 |
|  | Male | 1.48 (0.63, 3.46); p=0.37 | 0.63 (0.13, 2.98); p=0.56 | 0.81 (0.36, 1.82); p=0.60 |
|  | Female | 0.84 (0.28, 2.49); p=0.76 | 0.85 (0.23, 3.13); p=0.80 | 0.64 (0.26, 1.60); p=0.34 |
| MEHP (ng/ml) | All | 1.22 (0.61, 2.45); p=0.58 | 0.60 (0.20, 1.73); p=0.34 | 0.75 (0.39, 1.43); p=0.38 |
|  | Male | 1.24 (0.50, 3.12); p=0.64 | 0.31 (0.05, 2.01); p=0.22 | 0.85 (0.36, 2.00); p=0.72 |
|  | Female | 1.02 (0.32, 3.13); p=0.99 | 0.97 (0.25, 3.80); p=0.96 | 0.54 (0.20, 1.48); p=0.23 |
| MECPP (ng/mL) | All | 1.30 (0.60, 2.83); p=0.51 | 0.94 (0.28, 3.12); p=0.92 | 0.86 (0.41, 1.83); p=0.70 |
|  | Male | 1.43 (0.50, 4.10); p=0.50 | 1.00 (0.16, 6.15); p=0.99 | 0.55 (0.17, 1.84); p=0.33 |
|  | Female | 1.11 (0.31, 3.93); p=0.88 | 0.78 (0.15, 4.06); p=0.77 | 1.01 (0.35, 2.98); p=0.97 |
| ∑DiNP (ng/mL) | All | 1.32 (0.85, 2.07); p=0.22 | **2.14 (0.96, 4.06); p=0.047** | 1.13 (0.78, 1.64); p=0.52 |
|  | Male | 1.17 (0.66, 2.09); p=0.59 | 1.97 (0.67, 5.69); p=0.22 | 1.02 (0.61, 1.70); p=0.93 |
|  | Female | 1.87 (0.84, 4.16); p=0.12 | 2.32 (0.75, 7.19); p=0.14 | 1.21 (0.69, 2.14); p=0.51 |
| MiNP (ng/mL) | All | 1.48 (0.84, 2.59); p=0.17 | **2.82 (1.17, 6.86); p=0.022** | 1.09 (0.68, 1.74); p=0.73 |
|  | Male | 1.25 (0.61, 2.56); p=0.54 | 2.63 (0.75, 9.23); p=0.13 | 0.95 (0.50, 1.80); p=0.87 |
|  | Female | 2.38 (0.88, 6.30); p=0.088 | 3.15 (0.81, 12.24); p=0.097 | 1.22 (0.59, 2.54); p=0.59 |
| MCiOP (ng/mL) | All | 0.89 (0.27, 2.98); p=0.85 | 0.22 (0.02, 2.97); p=0.26 | 1.23 (0.48, 3.13); p=0.67 |
|  | Male | 1.08 (0.26, 4.42); p=0.91 | 0.80 (0.08 8.48); p=0.85 | 1.70 (0.60, 4.78); p=0.32 |
|  | Female | 0.64 (0.07, 6.37); p=0.71 | 0.01 (0.0001, 1.95); p=0.082 | 0.42 (0.05, 3.61); p=0.43 |

*Outcomes were modelled using multinomial logistic regression. Results were presented as relative risk ratios with no asthma ever (n=701, 386 males and 315 females) as the reference group (Total observations n=872, 484 males and 388 females). Models were adjusted for maternal asthma, birth weight for age z-scores, household income, maternal smoking, breastfeeding status, and maternal age.

‡MiBP*:* Mono-iso-butyl phthalate; MnBP: Mono-n-butyl phthalate; ∑MBP: Sum of MiBP and MnBP; MEHP: Mono-(2-ethyl-hexyl) phthalate; MECPP: Mono-(2-ethyl-5-carboxypentyl) phthalate; MiNP: mono-iso-nonyl phthalate; MCiOP: mono-carboxyiso-nonyl phthalate; ∑LMWP: molar sum of low molecular weight phthalates MEP, MiBP, MnBP and MHBP; MEP: Mono-ethyl phthalate; MHBP: Mono-(3-hydroxybutyl) phthalate; ∑HMWP : molar sum of MBzP, MEHP, MECPP, MCPP, MiNP, MCiOP and MiDP high molecular weight phthalates; MBzP: Mono-benzyl phthalate; MCPP: Mono-3-carboxypropyl phthalate; MiDP: Mono-iso-decyl phthalate; ∑DEHP: The sum of di-[2-ethylhexyl] phthalate metabolites; ∑DiNP: The sum of di-iso-nonyl phthalate metabolites

*Table S2. Associations between log-transformed BPA and phthalates‡ with spirometry outcomes in adjusted analyses in all participants.*

|  | FEV_1_ z-scores (Coefficient (95% CI); p-value) | | | FVC z-scores (Coefficient (95% CI); p-value) | | | FEV_1_/FVC z-scores (Coefficient (95% CI); p-value) | | |
| --- | --- | --- | --- | --- | --- | --- | --- | --- | --- |
|  | All | Male | Female | All | Male | Female | All | Male | Female |
| BPA (µg/L) | 0.005 (-0.02, 0.03); p=0.74 | -0.001 (-0.04, 0.04); p=0.96 | 0.02 (-0.03, 0.07); p=0.40 | 0.008 (-0.02, 0.04); p=0.61 | -0.002 (-0.04, 0.04); p=0.90 | 0.03 (-0.02, 0.07); p=0.29 | -0.003 (-0.04, 0.03); p=0.84 | -0.002 (-0.04, 0.04); p=0.93 | 0.005 (-0.05, 0.06); p=0.85 |
| ∑MBP (ng/mL) | **0.11 (0.04, 0.17); p=0.001** | 0.09 (-0.02, 0.20); p=0.10 | **0.12 (0.04, 0.21); p=0.005** | **0.09 (0.02, 0.16); p=0.011** | 0.05 (-0.06, 0.16); p=0.33 | **0.01 (0.03, 0.20); p=0.011** | **0.09 (0.003, 0.17); p=0.043** | 0.11 (-0.02, 0.24); p=0.10 | 0.06 (-0.05, 0.17); p=0.27 |
| MiBP (ng/ml) | **0.13 (0.05, 0.22); p=0.002** | 0.11 (-0.02, 0.24); p=0.10 | **0.15 (0.04, 0.26); p=0.008** | **0.09 (0.006, 0.18); p=0.036** | 0.08 (-0.05, 0.21); p=0.24 | 0.11 (-0.01, 0.22); p=0.075 | 0.08 (-0.02, 0.19); p=0.12 | 0.15 (-0.008, 0.30); p=0.064 | 0.01 (-0.13, 0.16); p=0.85 |
| MnBP (ng/mL) | **0.10 (0.03, 0.17); p=0.006** | 0.06 (-0.05, 0.18); p=0.28 | **0.12 (0.03, 0.21); p=0.007** | **0.08 (0.006, 0.15); p=0.033** | 0.02 (-0.09, 0.14); p=0.70 | **0.12 (0.03, 0.21); p=0.013** | **0.10 (0.01, 0.19); p=0.028** | 0.11 (-0.04, 0.25); p=0.14 | 0.09 (-0.03, 0.21); p=0.14 |
| ∑LMWP (ng/mL) | **0.08 (0.03, 0.14); p=0.004** | 0.07 (-0.03, 0.13); p=0.16 | **0.09 (0.02, 0.16); p=0.01** | **0.09 (0.03, 0.15); p=0.003** | 0.06 (-0.04, 0.15); p=0.27 | **0.12 (0.04, 0.19); p=0.002** | **0.08 (0.005, 0.15); p=0.036** | **0.14 (0.03, 0.26); p=0.015** | 0.03 (-0.07, 0.13); p=0.54 |
| MEP (ng/mL) | 0.04 (-0.01, 0.09); p=0.11 | 0.04 (-0.04, 0.12); p=0.35 | 0.05 (-0.02, 0.11); p=0.18 | 0.05 (-0.005, 0.10); p=0.078 | 0.02 (-0.06, 0.11); p=0.55 | **0.07 (0.002, 0.14); p=0.043** | 0.05 (-0.01, 0.11); p=0.13 | **0.10 (0.01, 0.19); p=0.025** | -0.003 (-0.09, 0.08); p=0.95 |
| MHBP (ng/mL) | **0.47 (0.12, 0.82); p=0.009** | **0.64 (0.08, 1.20); p=0.026** | 0.39 (-0.06, 0.84); p=0.09 | 0.35 (-0.01, 0.71); p=0.058 | 0.56 (-0.005, 0.12); p=0.052 | 0.25 (-0.22, 0.72); p=0.31 | 0.15 (-0.26, 0.55); p=0.47 | 0.06 (-0.60, 0.73); p=0.86 | 0.29 (-0.21, 0.80); p=0.25 |
| ∑HMWP (ng/mL) | -0.05 (-0.20, 0.10); p=0.53 | -0.14 (-0.35, 0.07); p=0.18 | 0.06 (-0.14, 0.27); p=0.55 | -0.04 (-0.19, 0.12); p=0.65 | -0.06 (-0.19, 0.07); p=0.34 | -0.04 (-0.19, 0.11); p=0.64 | 0.03 (-0.15, 0.21); p=0.72 | -0.04 (-0.30, 0.23); p=0.77 | 0.15 (-0.09, 0.40); p=0.23 |
| MBzP (ng/mL) | 0.12 (-0.04, 0.27); p=0.13 | -0.02 (-0.24, 0.19); p=0.82 | **0.30 (0.08, 0.53); p=0.008** | 0.06 (-0.09, 0.22); p=0.42 | -0.07 (-0.28, 0.15); p=0.54 | 0.05 (-0.17, 0.26); p=0.68 | 0.08 (-0.10, 0.27); p=0.37 | 0.04 (-0.21, 0.28); p=0.76 | 0.13 (-0.17, 0.43); p=0.40 |
| MCPP (ng/mL) | -0.06 (-0.29, 0.18); p=0.65 | 0.05 (-0.28, 0.39); p=0.76 | -0.19 (-0.53, 0.16); p=0.29 | -0.10 (-0.34, 0.15); p=0.44 | 0.07 (-0.26, 0.41); p=0.67 | -0.26 (-0.61, 0.10); p=0.16 | 0.21 (-0.06, 0.48); p=0.12 | 0.07 (-0.29, 0.43); p=0.69 | **0.50 (0.10, 0.90); p=0.015** |
| MiDP (ng/mL) | -0.06 (-0.18, 0.06); p=0.33 | -0.21 (-0.47, 0.05); p=0.12 | -0.004 (-0.14, 0.13); p=0.95 | -0.03 (-0.15, 0.09); p=0.66 | -0.22 (-0.48, 0.04); p=0.10 | 0.02 (-0.12, 0.17); p=0.73 | -0.04 (-0.19, 0.10); p=0.56 | -0.11 (-0.44, 0.23); p=0.54 | 0.005 (-0.16, 0.17); p=0.96 |
| ∑DEHP (ng/mL) | -0.06 (-0.20, 0.08); p=0.40 | -0.04 (-0.16, 0.07); p=0.46 | 0.02 (-0.17, 0.21); p=0.85 | -0.05 (-0.19, 0.10); p=0.52 | -0.11 (-0.33, 0.10); p=0.30 | 0.006 (-0.19, 0.21); p=0.95 | 0.05 (-0.11, 0.22); p=0.55 | 0.03 (-0.21, 0.27); p=0.79 | 0.09 (-0.14, 0.32); p=0.45 |
| MEHP (ng/ml) | -0.003 (-0.15, 0.15); p=0.97 | -0.14 (-0.36, 0.88); p=0.22 | 0.13 (-0.08, 0.34); p=0.21 | -0.04 (-0.19, 0.12); p=0.65 | -0.17 (-0.40, 0.05); p=0.13 | 0.09 (-0.13, 0.30); p=0.44 | 0.14 (-0.04, 0.33); p=0.12 | 0.09 (-0.18, 0.35); p=0.52 | 0.21 (-0.04, 0.46); p=0.099 |
| MECPP (ng/mL) | -0.14 (-0.31, 0.04); p=0.12 | -0.15 (-0.41, 0.12); p=0.29 | -0.15 (-0.39, 0.09); p=0.22 | -0.06 (-0.24, 0.12); p=0.54 | 0.02 (-0.25, 0.29); p=0.89 | -0.13 (-0.39, 0.12); p=0.30 | -0.08 (-0.29, 0.12); p=0.42 | -0.02 (-0.32, 0.29); p=0.92 | -0.14 (-0.43, 0.14); p=0.32 |
| ∑DiNP (ng/mL) | -0.001 (-0.09, 0.09); p=1.00 | -0.05 (-0.18, 0.08); p=0.44 | 0.05 (-0.08, 0.09); p=0.38 | -0.009 (-0.10, 0.08); p=0.84 | -0.10 (-0.32, 0.12); p=0.37 | -0.40 (-0.17, 0.09); p=0.53 | -0.02 (-0.13, 0.09); p=0.68 | -0.05 (-0.20, 0.10); p=0.52 | 0.02 (-0.13, 0.18); p=0.77 |
| MiNP (ng/mL) | 0.01 (-0.10, 0.13); p=0.80 | -0.06 (-0.23, 0.10); p=0.44 | 0.10 (-0.05, 0.26); p=0.20 | 0.004 (-0.11, 0.12); p=0.95 | -0.05 (-0.21, 0.12); p=0.57 | 0.07 (-0.09, 0.23); p=0.41 | -0.01 (-0.15, 0.13); p=0.87 | -0.06 (-0.25, 0.13); p=0.53 | 0.07 (-0.13, 0.27); p=0.50 |
| MCiOP (ng/mL) | -0.21 (-0.45, 0.02); p=0.072 | -0.15 (-0.45, 0.16); p=0.34 | -0.35 (-0.73, 0.03); p=0.07 | -0.23 (-0.47, 0.01); p=0.062 | -0.14 (-0.44, 0.17); p=0.37 | **-0.40 (-0.79, -0.002); p=0.049** | -0.28 (-0.56, 0.007); p=0.056 | -0.24 (-0.60, 0.12); p=0.20 | -0.36 (-0.83, 0.11); p=0.14 |

*Outcomes were assessed using linear mixed effects models to account for repeated measures. Models adjusted for household income, maternal smoking, breastfeeding status, and maternal age.

‡MiBP*:* Mono-iso-butyl phthalate; MnBP: Mono-n-butyl phthalate; ∑MBP: Sum of MiBP and MnBP; MEHP: Mono-(2-ethyl-hexyl) phthalate; MECPP: Mono-(2-ethyl-5-carboxypentyl) phthalate; MiNP: mono-iso-nonyl phthalate; MCiOP: mono-carboxyiso-nonyl phthalate; ∑LMWP: molar sum of low molecular weight phthalates MEP, MiBP, MnBP and MHBP; MEP: Mono-ethyl phthalate; MHBP: Mono-(3-hydroxybutyl) phthalate; ∑HMWP : molar sum of MBzP, MEHP, MECPP, MCPP, MiNP, MCiOP and MiDP high molecular weight phthalates; MBzP: Mono-benzyl phthalate; MCPP: Mono-3-carboxypropyl phthalate; MiDP: Mono-iso-decyl phthalate; ∑DEHP: The sum of di-[2-ethylhexyl] phthalate metabolites; ∑DiNP: The sum of di-iso-nonyl phthalate metabolites

*Table S3. Associations between log-transformed BPA and phthalates‡ with spirometry outcomes with lung function trajectory categories with additional confounders for sensitivity analysis.*

|  | | FEV_1_ (RRR (95% CI); p-value) | | FVC (RRR (95% CI); p-value) | | FEV_1_/FVC (RRR (95% CI); p-value) | | |
| --- | --- | --- | --- | --- | --- | --- | --- | --- |
|  |  | Low  (n=270, 161 males and 109 females) | Above average (n=34, 22 males and 12 females) | Low  (n=330, 200 males and 130 females | Above average (n=46, 27 males and 19 females | Very low (n=39, 25 males and 14 females) | Low-average (n=76, 36 males and 40 females) | Average-low (n=187, 119 males and 68 females) |
| BPA (µg/L) | All | 0.96 (0.90, 1.07); p=0.74 | 1.09 (0.89, 1.33); p=0.39 | 1.04 (0.94, 1.14); p=0.45 | 1.14 (0.95, 1.36); p=0.16 | 1.04 (0.88, 1.24); p=0.62 | 1.00 (0.88, 1.13); p=0.97 | 1.04 (0.95, 1.15); p=0.38 |
|  | Male | 1.04 (0.93, 1.16); p=0.48 | 1.11 (0.88, 1.40); p=0.37 | 1.07 (0.95, 1.20); p=0.27 | 1.10 (0.89, 1.37); p=0.38 | 0.98 (0.80, 1.20); p=0.87 | 0.91 (0.76, 1.08); p=0.26 | 1.02 (0.90, 1.14); p=0.78 |
|  | Female | 0.88 (0.75, 1.04); p=0.13 | 1.10 (0.70, 1.72); p=0.70 | 0.97 (0.81, 1.16); p=0.73 | 1.26 (0.84, 1.91); p=0.26 | 1.29 (0.81, 2.04); p=0.28 | 1.06 (0.93, 1.34); p=0.59 | 1.12 (0.93, 1.34); p=0.24 |
| ∑MBP (ng/mL) | All | 0.85 (0.67, 1.07); p=0.16 | 1.03 (0.65, 1.62); p=0.91 | 0.97 (0.75, 1.25); p=0.80 | 1.16 (0.76, 1.77); p=0.48 | 0.73 (0.47, 1.14); p=0.17 | 0.86 (0.61, 1.21); p=0.39 | 0.89 (0.69, 1.14); p=0.34 |
|  | Male | 0.85 (0.60, 1.21); p=0.38 | 0.73 (0.37, 1.47); p=0.39 | 1.30 (0.88, 1.94); p=0.18 | 1.29 (0.66, 2.50); p=0.46 | 0.65 (0.32, 1.32); p=0.23 | 1.06 (0.61, 1.86); p=0.82 | 0.91 (0.63, 1.32); p=0.63 |
|  | Female | 0.84 (0.61, 1.16); p=0.29 | 1.48 (0.72, 3.04); p=0.28 | 0.77 (0.54, 1.11); p=0.16 | 1.15 (0.63, 2.08); p=0.65 | 0.71 (0.37, 1.36); p=0.31 | 0.74 (0.47, 1.16); p=0.19 | 0.88 (0.61, 1.26); p=0.48 |
| MiBP (ng/ml) | All | 0.88 (0.66, 1.17); p=0.37 | 0.93 (0.52, 1.66); p=0.81 | 0.99 (0.72, 1.34); p=0.96 | 1.12 (0.66, 1.90); p=0.67 | 0.65 (0.37, 1.15); p=0.14 | 1.11 (0.73, 1.69); p=0.63 | 0.96 (0.70, 1.31); p=0.80 |
|  | Male | 0.90 (0.59, 1.35); p=0.60 | 0.85 (0.38, 1.87); p=0.68 | 1.39 (0.87, 2.21); p=0.16 | 1.83 (0.86, 3.93); p=0.12 | 0.70 (0.32, 1.55); p=0.38 | 1.42 (0.75, 2.70); p=0.29 | 0.92 (0.59, 0.43); p=0.71 |
|  | Female | 0.85 (0.56, 1.28); p=0.44 | 1.03 (0.40, 2.67); p=0.95 | 0.73 (0.46, 1.14); p=0.17 | 0.77 (0.35, 1.72); p=0.53 | 0.52 (0.20, 1.32); p=0.17 | 0.92 (0.52, 1.64); p=0.79 | 1.05 (0.65, 1.68); p=0.85 |
| MnBP (ng/mL) | All | 0.83 (0.65, 1.07); p=0.16 | 1.08 (0.68, 1.74); p=0.74 | 0.98 (0.74, 1.28); p=0.87 | 1.22 (0.79, 1.89); p=0.37 | 0.69 (0.42, 1.13); p=0.14 | 0.77 (0.53, 1.13); p=0.19 | 0.86 (0.66, 1.12); p=0.27 |
|  | Male | 0.85 (0.59, 1.24); p=0.41 | 0.74 (0.35, 1.56); p=0.43 | 1.29 (0.85, 1.98); p=0.23 | 1.15 (0.56, 2.37); p=0.71 | 0.55 (0.24, 1.24); p=0.15 | 0.94 (0.51, 1.73); p=0.85 | 0.95 (0.66, 1.41); p=0.82 |
|  | Female | 0.82 (0.58, 1.16); p=0.27 | 1.68 (0.81, 3.48); p=0.16 | 0.79 (0.54, 1.16); p=0.23 | 1.37 (0.74, 2.52); p=0.32 | 0.74 (0.37, 1.48); p=0.39 | 0.66 (0.40, 1.08); p=0.10 | 0.78 (0.53, 1.16); p=0.23 |
| ∑LMWP (ng/mL) | All | 0.92 (0.75, 1.12); p=0.42 | 1.14 (0.74, 1.74); p=0.55 | 0.97 (0.77, 1.22); p=0.80 | 1.22 (0.82, 1.81); p=0.32 | 0.89 (0.60, 1.30); p=0.54 | 0.91 (0.67, 1.24); p=0.55 | 0.92 (0.74, 1.14); p=0.44 |
|  | Male | 0.86 (0.61, 1.12); p=0.36 | 0.91 (0.50, 1.64); p=0.75 | 1.16 (0.81, 1.66); p=0.41 | 1.34 (0.75, 2.41); p=0.33 | 0.60 (0.32, 1.10); p=0.10 | 1.23 (0.72, 2.10); p=0.45 | 0.90 (0.64, 1.27); p=0.55 |
|  | Female | 0.96 (0.73, 1.25); p=0.75 | 1.41 (0.68, 2.91); p=0.35 | 0.86 (0.63, 1.18); p=0.36 | 1.14 (0.64, 2.03); p=0.66 | 1.19 (0.64, 2.20); p=0.58 | 0.84 (0.58, 1.23); p=0.37 | 0.95 (0.70, 1.29); p=0.75 |
| MEP (ng/mL) | All | 0.95 (0.80, 1.12); p=0.53 | 1.05 (0.74, 1.49); p=0.79 | 0.96 (0.80, 1.16); p=0.70 | 1.15 (0.83, 1.60); p=0.39 | 1.05 (0.76, 1.45); p=0.75 | 1.05 (0.81, 1.35); p=0.81 | 0.96 (0.80, 1.16); p=0.68 |
|  | Male | 0.93 (0.72, 1.20); p=0.57 | 1.09 (0.69, 1.72); p=0.72 | 1.01 (0.77, 1.35); p=0.93 | 1.36 (0.85, 2.18); p=0.20 | 0.76 (0.48, 1.19); p=0.22 | 1.36 (0.88, 2.10); p=0.16 | 0.95 (0.73, 1.23); p=0.70 |
|  | Female | 0.95 (0.75, 1.21); p=0.69 | 0.91 (0.51, 1.62); p=0.74 | 0.92 (0.70, 1.20); p=0.53 | 0.95 (0.59, 1.53); p=0.84 | 1.65 (0.95, 2.87); p=0.074 | 0.99 (0.71, 1.39); p=0.97 | 1.00 (0.76, 1.32); p=1.00 |
| MHBP (ng/mL) |  | **0.24 (0.07, 0.74); p=0.014** | **5.95 (1.06, 33.35); p=0.042** | **0.19 (0.06, 0.60); p=0.005** | 1.51 (0.28, 8.23); p=0.63 | 0.41 (0.05, 3.31); p=0.40 | 0.76 (0.16, 3.60); p=0.73 | 0.38 (0.11, 1.30); p=0.12 |
|  | Male | 0.24 (0.04, 1.57); p=0.14 | 3.87 (0.33, 45.18); p=0.28 | 0.27 (0.04, 1.69); p=0.16 | 0.84 (0.07, 12.87); p=0.90 | 0.13 (0.002, 9.40); p=0.35 | 0.85 (0.05, 16.03); p=0.92 | 1.62 (0.27, 9.68); p=0.60 |
|  | Female | **0.19 (0.04, 0.92); p=0.038** | **19.15 (1.06, 348.46); p=0.046** | **0.11 (0.02, 0.57); p=0.008** | 1.41 (0.16, 12.10); p=0.77 | 0.44 (0.03, 6.69); p=0.55 | 0.29 (0.04, 2.29); p=0.24 | **0.11 (0.02, 0.68); p=0.018** |
| ∑HMWP (ng/mL) | All | 0.82 (0.51, 1.33); p=0.42 | 1.52 (0.56, 4.16); p=0.41 | 0.73 (0.43, 1.24); p=0.25 | 1.22 (0.48, 3.09); p=0.67 | 1.08 (0.44, 2.65); p=0.86 | 0.99 (0.49, 2.05); p=0.99 | 0.98 (0.58, 1.66); p=0.95 |
|  | Male | 0.83 (0.40, 1.70); p=0.60 | 1.30 (0.34, 4.92); p=0.70 | 0.69 (0.32, 1.50); p=0.35 | 1.03 (0.27, 4.03); p=0.96 | 1.29 (0.36, 4.56); p=0.70 | 1.78 (0.52, 6.06); p=0.36 | 1.50 (0.70, 3.02); p=0.30 |
|  | Female | 0.69 (0.35, 1.37); p=0.29 | 2.32 (0.40. 13.56); p=0.35 | 0.68 (0.31, 1.50); p=0.34 | 1.43 (0.35, 5.80); p=0.62 | 1.03 (0.24, 4.38); p=0.97 | 0.78 (0.30, 2.07); p=0.62 | 0.68 (0.31, 1.49); p=0.34 |
| MBzP (ng/mL) | All | 0.64 (0.36, 1.12); p=0.12 | 0.73 (0.27, 2.02); p=0.55 | 1.00 (0.55, 1.82); p=1.00 | 1.43 (0.63, 3.24); p=0.39 | 0.63 (0.20, 1.93); p=0.42 | 0.90 (0.40, 2.03); p=0.80 | 0.83 (0.47, 1.46); p=0.52 |
|  | Male | 0.52 (0.22, 1.22); p=0.13 | 0.31 (0.05, 2.15); p=0.24 | 1.10 (0.51, 2.38); p=0.80 | 1.49 (0.49, 4.55); p=0.48 | 0.61 (0.12, 3.21); p=0.56 | 1.09 (0.40, 3.00); p=0.87 | 1.13 (0.54, 2.33); p=0.75 |
|  | Female | 0.76 (0.32, 1.81); p=0.54 | 1.79 (0.38, 8.37); p=0.46 | 0.86 (0.33, 2.26); p=0.75 | 1.48 (0.35, 6.19); p=0.59 | 0.47 (0.08, 2.71); p=0.40 | 0.45 (0.12, 1.60); p=0.22 | 0.57 (0.22, 1.49); p=0.25 |
| MCPP (ng/mL) | All | 0.86 (0.40, 1.82); p=0.69 | 1.78 (0.48, 6.58); p=0.39 | 0.66 (0.30, 1.45); p=0.30 | 0.62 (0.15, 2.58); p=0.51 | 0.58 (0.13, 2.64); p=0.49 | 0.67 (0.21, 2.14); p=0.50 | 0.75 (0.34, 1.68); p=0.49 |
|  | Male | 0.71 (0.26, 1.97); p=0.52 | 1.11 (0.22, 5.70); p=0.90 | 0.51 (0.19, 1,42); p=0.20 | 0.17 (0.02, 1.40); p=0.10 | 0.23 (0.02, 2.28); p=0.21 | 0.32 (0.04, 2.43); p=0.27 | 1.19 (0.44, 3.25); p=0.73 |
|  | Female | 0.83 (0.25, 2.71); p=0.76 | 2.35 (0.19, 28.57); p=0.50 | 1.01 (0.27, 3.76); p=0.99 | 1.96 (0.23, 16.45); p=0.54 | 0.94 (0.12, 7.48); p=0.96 | 0.81 (0.17, 3.94); p=0.80 | **0.20 (0.04, 0.97); p=0.046** |
| MiDP (ng/mL) | All | 1.14 (0.78, 1.66); p=0.50 | 0.92 (0.39, 2.15); p=0.85 | 1.46 (0.94, 2.26); p=0.093 | 1.25 (0.59, 2.64); p=0.56 | 1.04 (0.53, 2.03); p=0.91 | 1.12 (0.65, 1.93); p=0.69 | 0.96 (0.64, 1.46); p=0.87 |
|  | Male | 1.31 (0.55, 3.32); p=0.55 | 1.71 (0.36, 8.19); p=0.50 | 1.90 (0.67, 5.38); p=0.23 | 2.45 (0.46, 13.43); p=0.30 | 2.09 (0.53, 8.27); p=0.29 | 1.64 (0.43, 6.25); p=0.47 | 0.74 (0.29, 1.91); p=0.54 |
|  | Female | 1.12 (0.71, 1.76); p=0.60 | 0.95 (0.29, 3.13); p=0.94 | 1.31 (0.78, 2.19); p=0.30 | 1.06 (0.41, 2.74); p=0.90 | 1.03 (0.42, 2.51); p=0.95 | 0.99 (0.52, 1.89); p=0.98 | 1.13 (0.68, 1.89); p=0.63 |
| ∑DEHP (ng/mL) | All | 0.84 (0.54, 1.31); p=0.44 | 1.21 (0.51, 2.87); p=0.66 | 0.71 (0.44, 1.16); p=0.17 | 1.01 (0.45, 2.28); p=0.98 | 1.12 (0.49, 2.55); p=0.79 | 0. 92 (0.47, 1.79); p=0.80 | 1.00 (0.62, 1.63); p=0.98 |
|  | Male | 0.88 (0.46, 1.69); p=0.70 | 1.02 (0.32, 3.31); p=0.97 | 0.73 (0.37, 1.47); p=0.39 | 0.79 (0.23, 2.66); p=0.71 | 1.14 (0.36, 3.63); p=0.83 | 1.09 (0.37, 3.22); p=0.87 | 1.18 (0.59, 2.33); p=0.64 |
|  | Female | 0.67 (0.35, 1.30); p=0.24 | 1.69 (0.42, 6.92); p=0.48 | 0.67 (0.35, 1.30); p=0.24 | 1.69 (0.41, 6.96); p=0.47 | 1.29 (0.37, 4.51); p=0.69 | 0.98 (0.40, 2.41); p=0.97 | 0.84 (0.40, 1.75); p=0.64 |
| MEHP (ng/ml) | All | 0.86 (0.52, 1.41); p=0.55 | 1.31 (0.50, 3.44); p=0.58 | 0.80 (0.47, 1.38); p=0.43 | 1.21 (0.49, 2.92); p=0.68 | 0.68 (0.27, 1.76); p=0.43 | 0.62 (0.29, 1.33); p=0.22 | 1.06 (0.62, 1.82); p=0.83 |
|  | Male | 0.88 (0.43, 1.79); p=0.73 | 0.98 (0.27, 3.56); p=0.97 | 0.83 (0.39, 1.77); p=0.63 | 0.77 (0.20, 2.95); p=0.70 | 0.62 (0.16, 2.38); p=0.49 | 0.73 (0.22, 2.43); p=0.61 | 1.42 (0.67, 2.98); p=0.36 |
|  | Female | 0.70 (0.33, 1.47); p=0.34 | 3.44 (0.58, 20.40), p=0.19 | 0.70 (0.31, 1.60); p=0.39 | 2.09 (0.51, 8.51); p=0.30 | 0.97 (0.22, 4.19); p=0.96 | 0.58 (0.20, 1.66); p=0.31 | 0.70 (0.30, 1.63); p=0.41 |
| MECPP (ng/mL) | All | 0.86 (0.50, 1.46); p=0.57 | 0.68 (0.22, 2.12); p=0.51 | 0.72 (0.40, 1.27); p=0.26 | 0.73 (0.27, 2.02); p=0.55 | 1.63 (0.63, 4.21); p=0.32 | 1.52 (0.70, 3.32); p=0.29 | 1.03 (0.57, 0.86); p=0.92 |
|  | Male | 0.81 (0.37, 1.79); p=0.61 | 0.45 (0.09, 2.37); p=0.35 | 0.65 (0.28, 1.48); p=0.30 | 0.38 (0.07, 2.02); p=0.25 | 1.77 (0.40, 6.80); p=0.40 | 1.93 (0.57, 6.52); p=0.29 | 1.02 (0.43, 2.44); p=0.97 |
|  | Female | 0.80 (0.37, 1.75); p=0.58 | 0.60 (0.08, 4.43); p=0.62 | 0.81 (0.34, 1.93); p=0.63 | 1.18 (0.29, 5.00); p=0.82 | 1.32 (0.28, 6.24); p=0.72 | 1.90 (0.66, 5.47); p=0.23 | 1.09 (0.43, 2.73); p=0.86 |
| ∑DiNP (ng/mL) | All | 0.93 (0.70, 1.23); p=0.62 | 1.47 (0.77, 2.85); p=0.25 | 0.82 (0.60, 1.13); p=0.23 | 1.23 (0.68, 2.22); p=0.50 | 1.16 (0.67, 2.00); p=0.60 | 1.06 (0.69, 1.63); p=0.79 | 1.00 (0.74, 1.36); p=0.99 |
|  | Male | 0.91 (0.61, 1.37); p=0.66 | 1.33 (0.58, 3.01); p=0.50 | 0.76 (0.48, 1.20); p=0.24 | 1.27 (0.53, 3.03); p=0.59 | 1.43 (0.66, 3.06); p=0.36 | 1.72 (0.83, 3.61); p=0.15 | 1.28 (0.83, 1.97); p=0.26 |
|  | Female | 0.92 (0.62, 1.37); p=0.68 | 1.58 (0.48, 5.17); p=0.45 | 0.89 (0.56, 1.42); p=0.63 | 1.05 (0.45, 2.47); p=0.91 | 0.82 (0.35, 1.95); p=0.66 | 0.84 (0.47, 1.48); p=0.54 | 0.83 (0.52, 1.31); p=0.41 |
| MiNP (ng/mL) | All | 0.90 (0.63, 1.29); p=0.58 | 1.59 (0.72, 3.50); p=0.25 | 0.81 (0.54, 1.20); p=0.29 | 1.28 (0.63, 2.63); p=0.49 | 1.09 (0.55, 2.15); p=0.81 | 1.06 (0.62, 1.84); p=0.82 | 0.93 (0.63, 1.37); p=0.71 |
|  | Male | 0.92 (0.55, 1.52); p=0.74 | 0.48 (0.55, 4.01); p=0.44 | 0.76 (0.44, 1.31); p=0.32 | 1.45 (0.51, 4.11); p=0.48 | 1.40 (0.56, 3.52); p=0.47 | 1.85 (0.77, 4.48); p=0.17 | 1.28 (0.75, 2.18); p=0.37 |
|  | Female | 0.86 (0.51, 1.45); p=0.57 | 1.66 (0.40, 6.90); p=0.49 | 0.87 (0.48, 1.57); p=0.64 | 0.98 (0.34, 2.81); p=0.97 | 0.65 (0.22, 1.92); p=0.43 | 0.66 (0.32, 1.36); p=0.26 | 0.65 (0.36, 1.17); p=0.15 |
| MCiOP (ng/mL) | All | 0.72 (0.34, 1.52); p=0.39 | 0.73 (0.15, 3.55); p=0.69 | 0.54 (0.24, 1.20); p=0.13 | 0.50 (0.10, 2.55); p=0.41 | 1.72 (0.43, 6.86); p=0.44 | 1.41 (0.43, 4.61); p=0.57 | 1.93 (0.81, 4.64); p=0.14 |
|  | Male | 0.62 (0.24, 1.59); p=0.31 | 0.51 (0.05, 4.97); p=0.56 | **0.35 (0.12, 0.99); p=0.049** | 0.16 (0.01, 2.48); p=0.19 | 1.62 (0.22, 12.03); p=0.64 | 1.41 (0.20, 10.06); p=0.73 | 2.64 (0.82, 8.45); p=0.10 |
|  | Female | 0.78 (0.20, 3.01); p=0.72 | 0.90 (0.04, 19.01); p=0.95 | 1.40 (0.28, 7.14); p=0.68 | 2.61 (0.22, 30.91); p=0.45 | 2.19 (0.21, 23.35); p=0.52 | 2.31 (0.38, 14.21); p=0.37 | 0.64 (0.11, 3.85); p=0.63 |

*Outcomes were modelled using multinomial logistic regression. Results were presented as relative risk ratios with the average trajectory (FEV_1_: n=223, 137 male and 86 females; FVC: n=151, 93 males and 58 females; FEV_1_/FVC: n=225 males, 140 males and 85 females) as the reference group. Models adjusted for household income, maternal smoking, breastfeeding status, and maternal age.

‡MiBP*:* Mono-iso-butyl phthalate; MnBP: Mono-n-butyl phthalate; ∑MBP: Sum of MiBP and MnBP; MEHP: Mono-(2-ethyl-hexyl) phthalate; MECPP: Mono-(2-ethyl-5-carboxypentyl) phthalate; MiNP: mono-iso-nonyl phthalate; MCiOP: mono-carboxyiso-nonyl phthalate; ∑LMWP: molar sum of low molecular weight phthalates MEP, MiBP, MnBP and MHBP; MEP: Mono-ethyl phthalate; MHBP: Mono-(3-hydroxybutyl) phthalate; ∑HMWP : molar sum of MBzP, MEHP, MECPP, MCPP, MiNP, MCiOP and MiDP high molecular weight phthalates; MBzP: Mono-benzyl phthalate; MCPP: Mono-3-carboxypropyl phthalate; MiDP: Mono-iso-decyl phthalate; ∑DEHP: The sum of di-[2-ethylhexyl] phthalate metabolites; ∑DiNP: The sum of di-iso-nonyl phthalate metabolites.

**Supplementary Figures**

**
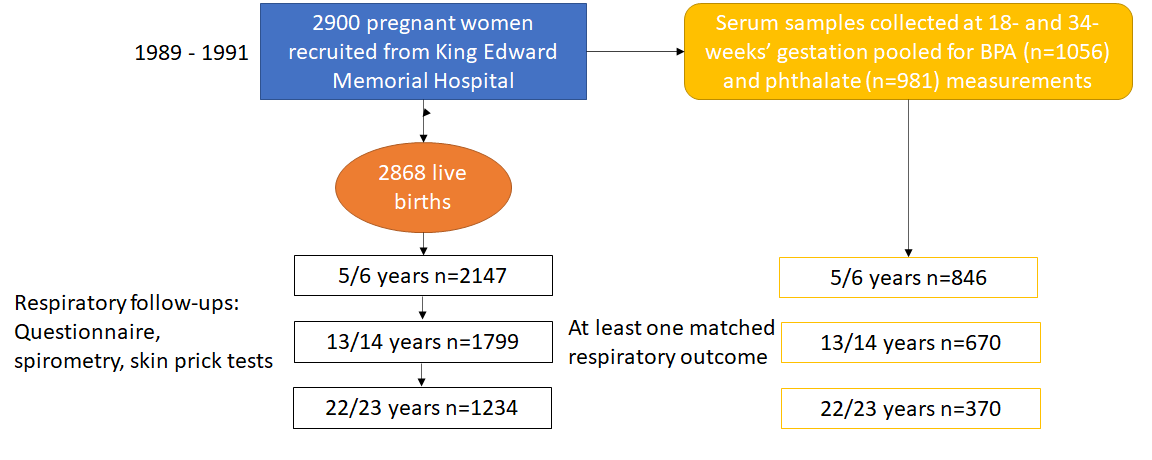
**

Figure S1. Flowchart of Raine Study participants included in this study. The Raine Study has published findings showing no selection bias in the cohort after 20 years of follow-up.^12^


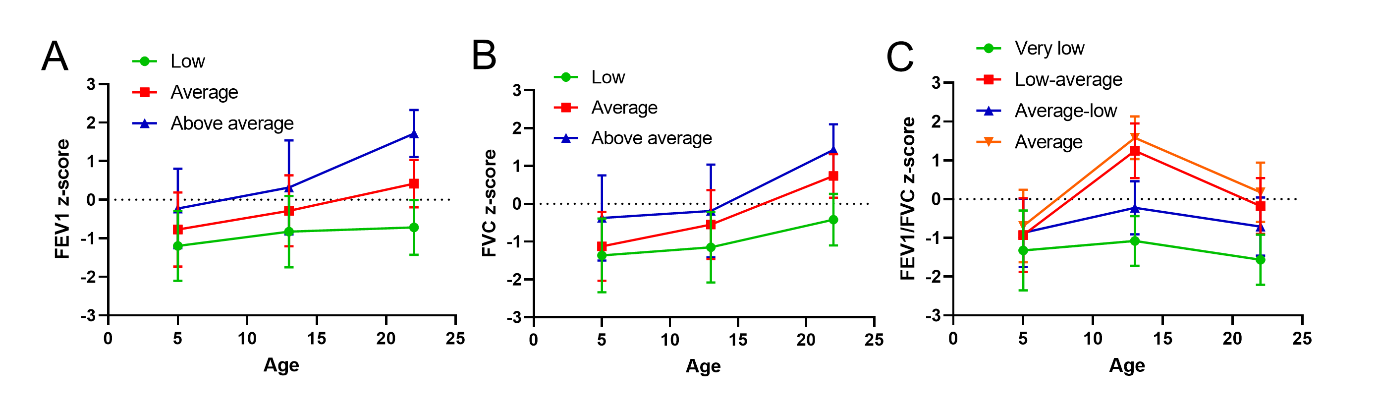


Figure S2. Lung function trajectories for FEV_1_ (A), FVC (B) and FEV_1_/FVC (C) z-scores of Raine Study participants from childhood to adulthood derived from group-based trajectory modelling.


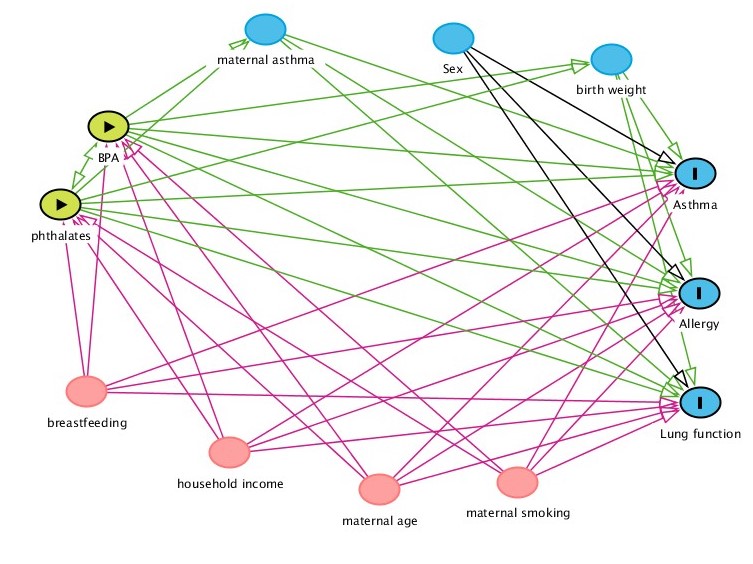


Figure S3. Direct Acyclic Graph (DAG) to identify the minimal sufficient adjustment sets for estimating the total effect of BPA and phthalates on respiratory outcomes – asthma, allergies and lung function.


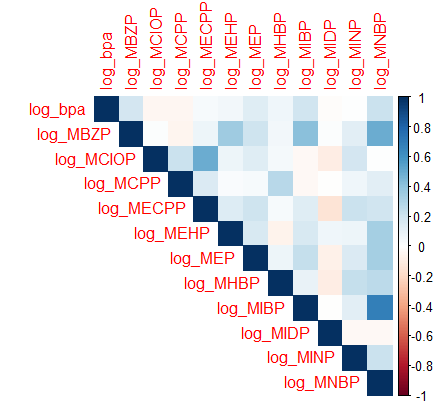


Figure S4. Correlation plot of log transformed BPA and phthalate metabolites.


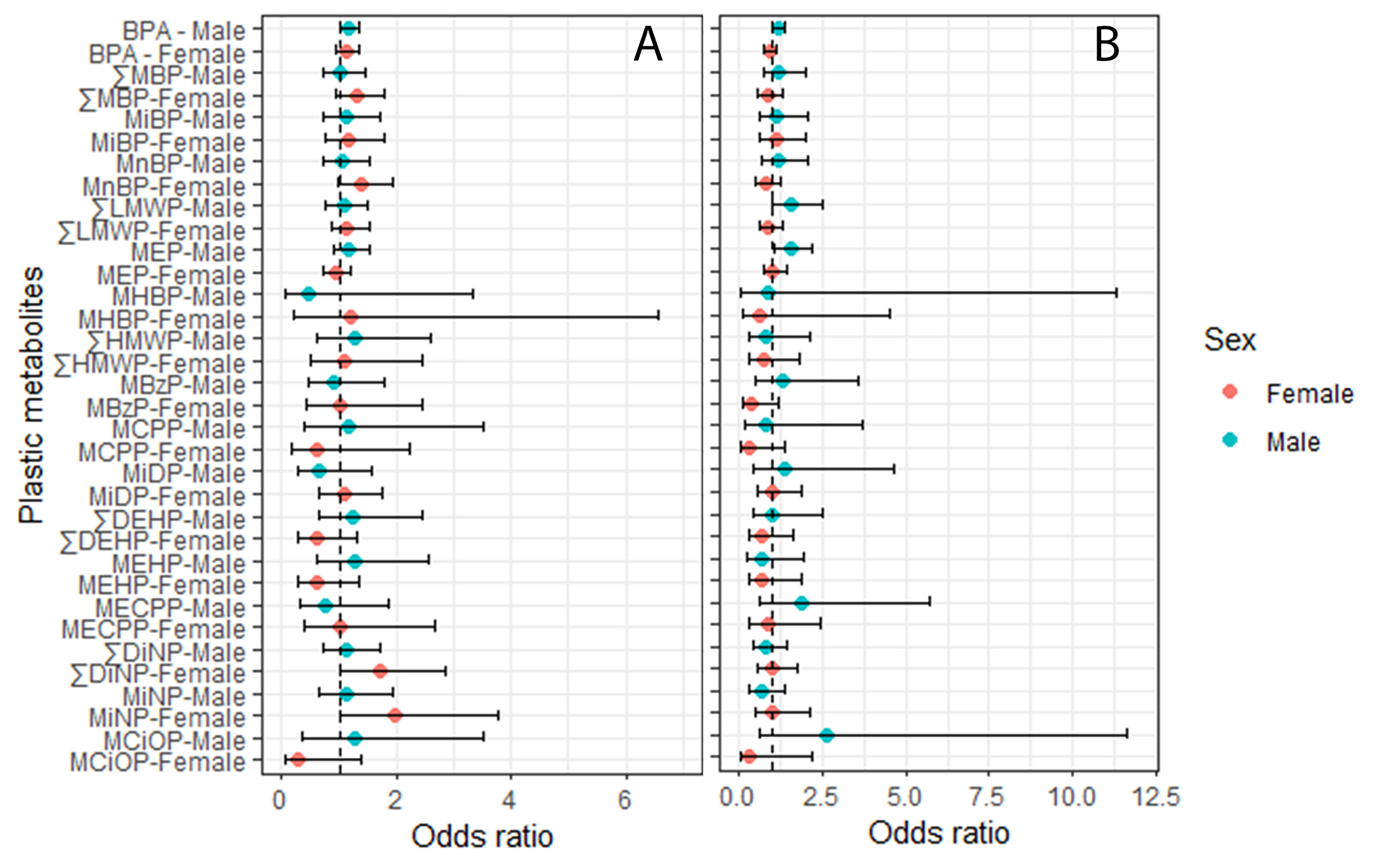


Figure S5. Forest plots for the odds ratio for asthma (A) and allergy (B) measured by skin prick tests with prenatal bisphenol A (BPA) and phthalates exposure in offspring at 5, 13 and 22-years of age.

**References**

1 Newnham JP, Evans SF, Michael CA, Stanley FJ, Landau LI. Effects of frequent ultrasound during pregnancy: a randomised controlled trial. The Lancet. 1993; **342**: 887-91.

2 Miller MR, Hankinson J, Brusasco V, Burgos F, Casaburi R, Coates A, Crapo R, Enright P, van der Grinten CPM, Gustafsson P, Jensen R, Johnson DC, MacIntyre N, McKay R, Navajas D, Pedersen OF, Pellegrino R, Viegi G, Wanger J. Standardisation of spirometry. Eur Respir J. 2005; **26**: 319-38.

3 Quanjer PH, Stanojevic S, Cole TJ, Baur X, Hall GL, Culver BH, Enright PL, Hankinson JL, Ip MSM, Zheng J, Stocks J, Initiative tEGLF. Multi-ethnic reference values for spirometry for the 3–95-yr age range: the global lung function 2012 equations. Eur Respir J. 2012; **40**: 1324-43.

4 Hall G, Thompson B, Stanojevic S, Abramson M, Beasley R, Coates A, Dent A, Eckert B, James A, Filsel S, Musk A, Nolan G, Dixon B, O'Dea C, Savage J, Stocks J, Swanney M. The Global Lung Initiative 2012 reference values reflect contemporary Australasian spirometry. Respirology. 2012; **17**: 1150-1.

5 Textor J, van der Zander B, Gilthorpe MS, Liśkiewicz M, Ellison GT. Robust causal inference using directed acyclic graphs: the R package ‘dagitty’. Int J Epidemiol. 2017; **45**: 1887-94.

6 WHO Multi Centre Growth Reference Study Group, de Onis M. WHO Child Growth Standards based on length/height, weight and age. Acta Paediatr. 2006; **95**: 76-85.

7 Sanna F, Locatelli F, Sly PD, White E, Blake D, Heyworth J, Hall GL, Foong RE. Characterization of lung function trajectories and associated early life predictors in an Australian birth cohort study. ERJ Open Research. 2022: 00072-2022.

8 Jones BL, Nagin DS. A note on a Stata plugin for estimating group-based trajectory models. Sociological Methods & Research. 2013; **2013**: 608-13.

9 de Jong VMT, Eijkemans MJC, van Calster B, Timmerman D, Moons KGM, Steyerberg EW, van Smeden M. Sample size considerations and predictive performance of multinomial logistic prediction models. Stat Med. 2019; **38**: 1601-19.

10 Carrico C, Gennings C, Wheeler DC, Factor-Litvak P. Characterization of Weighted Quantile Sum Regression for Highly Correlated Data in a Risk Analysis Setting. J Agric Biol Environ Stat. 2015; **20**: 100-20.

11 Wasserstein RL, Lazar NA. The ASA Statement on p-Values: Context, Process, and Purpose. The American Statistician. 2016; **70**: 129-33.

12 White SW, Eastwood PR, Straker LM, Adams LA, Newnham JP, Lye SJ, Pennell CE. The Raine study had no evidence of significant perinatal selection bias after two decades of follow up: a longitudinal pregnancy cohort study. BMC Pregnancy Childbirth. 2017; **17**: 207.
