# Supplementary material for: Longitudinal effects of prenatal exposure to plastic‐derived chemicals and their metabolites on asthma and lung function from childhood into adulthood
Source: Respirology. 2022 Oct 2;28(3):236–46. doi: 10.1111/resp.14386 (PMC10946907; doi:10.1111/resp.14386)
Supplement: Supplementary file 2 — Visual Abstract Longitudinal effects of prenatal exposure to plastic‐derived chemicals and their metabolites on asthma and lung function from childhood into adulthood [file RESP-28-236-s002.pdf]

Longitudinal effects of prenatal exposure to plastic-derived chemicals and their metabolites on asthma and lung function from childhood into adulthood

EDITOR'S CHOICE

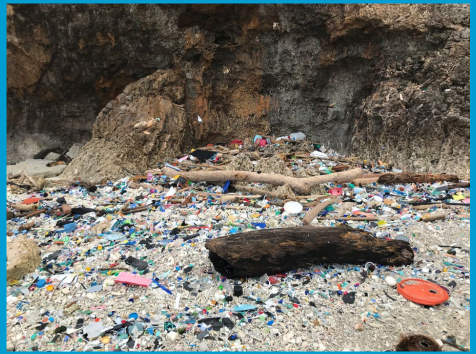

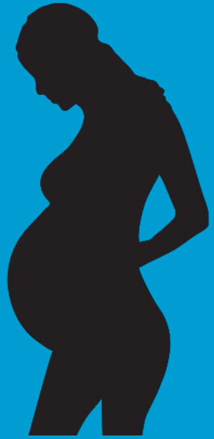

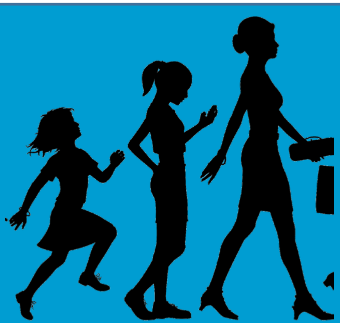

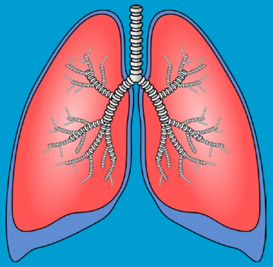

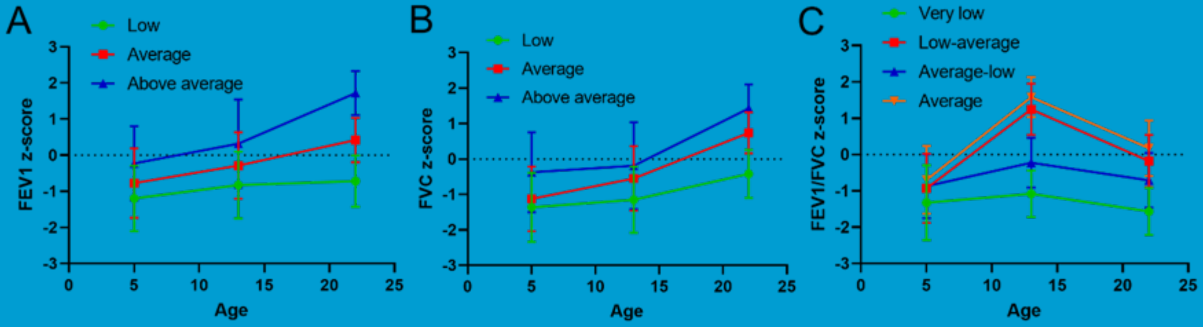

This study aimed to assess the effects of exposure to plastic by-products and their metabolites in pregnancy on asthma, allergy and lung function in childhood, adolescence, and adulthood, and on asthma phenotypes and lung function trajectories.

Results suggest that prenatal exposure to prenatal bisphenol A and phthalates affect asthma risk, particularly in males, however lung function was not adversely affected.

Respirology

Longitudinal effects of prenatal exposure to plastic-derived chemicals and their metabolites on asthma and lung function from childhood into adulthood
Foong et al. Respirology 2022; DOI:10.111/resp.14386
